# Harnessing exDNA for precision exatecan delivery in cancer: a novel antibody-drug conjugate approach

**DOI:** 10.1186/s12943-025-02462-z

**Published:** 2025-10-13

**Authors:** Zaira Ianniello, Huimei Lu, Elias Quijano, Daniel A. Colón-Ríos, Madison Rackear, Venu Bommireddy, Dale L. Ludwig, Zhiyuan Shen, Peter M. Glazer

**Affiliations:** 1 Department of Therapeutic Radiology, Yale University School of Medicine, New Haven, CT 06520 USA; 2 Rutgers Cancer Institute, New Brunswick, NJ 08901 USA; 3 Department of Genetics, Yale University School of Medicine, New Haven, CT 06520 USA; 4 Gennao Bio, Hopewell, NJ 08525 USA

**Keywords:** Antibody-Drug conjugate, Drug delivery system, ExDNA, Tumor microenvironment, DNA damage, Topoisomerase-I, ENT2, Targeted cancer therapy, Nuclear penetration, BRCA-deficient tumors

## Abstract

**Background:**

Current antibody-drug conjugates (ADCs) face limitations due to a lack of tumor-selective targets, inefficient internalization, and challenges in reaching tumors in challenging sites, ultimately limiting their therapeutic efficacy. We developed and characterized V66-exatecan, a novel ADC composed of V66, a humanized antibody with high affinity for extracellular DNA (exDNA), conjugated to exatecan via a cleavable linker. This ADC employs a dual-targeting mechanism based on exDNA and ENT2 transporter expression to enhance nuclear drug delivery and tumor specificity. This study evaluates its anti-tumor activity, mechanism of action, ability to treat challenging tumors, and safety profile.

**Methods:**

To validate tumor selectivity, V66 or a control antibody were conjugated to a fluorescent tag and injected intravenously into tumor-bearing mice; biodistribution analysis demonstrated selective accumulation in tumors and nuclear localization within tumor cells. V66 was then conjugated to exatecan via a cleavable linker. In vitro assays across diverse cancer cell lines assessed cytotoxicity, DNA damage response (DDR) activation, and TOP1 degradation. In vivo efficacy was evaluated in xenograft models of triple-negative breast cancer (TNBC) and BRCA1/2-deficient tumors, including intracranial medulloblastoma. These models were used to assess tumor growth inhibition, survival benefit, and blood-brain barrier (BBB) permeability. Toxicity was assessed through a dose-escalation study, with analysis of hematologic parameters, histopathology of major organs, and liver and kidney function tests (ALT, AST, BUN, total protein) following short- and long-term treatment.

**Results:**

V66-exatecan demonstrated potent anti-tumor activity in multiple cancer cell lines but not on healthy mouse primary fibroblasts, with EC_50_ values in the low nanomolar range. It induced robust DDR signaling, TOP1 degradation, and bystander killing effects. BRCA1/2-deficient models exhibited enhanced penetration and sensitivity, with up to 17-fold lower EC_50_ compared to BRCA-proficient controls. In vivo, V66-exatecan significantly inhibited tumor growth and extended survival in both TNBC and BRCA-mutant CNS tumors, including complete regressions and prolonged median survival in BRCA2-deficient models. Toxicology studies revealed no significant hematologic, renal, hepatic, or bone marrow toxicity, even at high or repeated doses.

**Conclusions:**

V66-exatecan represents a next-generation of ADCs that overcomes key limitations of traditional platforms by exploiting exDNA-driven tumor selectivity and ENT2-mediated nuclear delivery. It demonstrates broad therapeutic efficacy and a favorable safety profile, supporting its potential for treating DDR-deficient and hard-to-reach tumors.

**Supplementary Information:**

The online version contains supplementary material available at 10.1186/s12943-025-02462-z.

## Background

Antibody-drug conjugates (ADCs) offer a potential realization of Paul Ehrlich’s “magic bullet” concept, which envisioned a targeted approach to drug delivery that selectively destroys harmful cells while sparing healthy ones [1, 2]. By combining the specificity of monoclonal antibodies with potent cytotoxic drugs, ADCs deliver therapeutic agents directly to tumor cells, minimizing off-target effects and enhancing treatment efficacy [3, 4]. With over 10 approved drugs and numerous others in clinical development, ADCs are considered a revolution in cancer treatment [5]. The conventional mechanism of an ADC relies on targeting specific tumor antigens, leading to endosomal internalization and release of a cytotoxic payload that disrupts critical cellular components, such as microtubules or DNA, resulting in cell death [6].

Several factors limit the effectiveness of ADCs. One key challenge is the requirement for tumor antigens to be expressed at much higher levels on cancer cells than on healthy tissues, to provide for selective targeting while minimizing damage to normal cells [7, 8]. However, the limited set of tumor antigens that can be effectively targeted restricts the broader applicability of ADCs. Some antigens are not universally expressed across all cancer types, and others may also be present on normal tissues, leading to undesirable off-target effects [9]. Another major limitation is ADC internalization via endocytosis or macropinocytosis [10], which, upon fusion with lysosomes, results in acidic conditions that break down the linkers and release the cytotoxic payload [11–13]. Since only 1%−2% of the administered ADC dose reaches tumor cells [14], selecting a highly potent cytotoxin is essential to ensure effective therapeutic outcomes even with limited delivery efficiency.

To address the challenge of limited antigen availability for targeted therapies, the tumor microenvironment offers an additional pool of potential targets, including extracellular DNA (exDNA) released from necrotic tumor cells. As a mechanism of reparative cell death, necrosis plays a key role in tumor progression, as exDNA sustains inflammation and reshapes the tumor microenvironment, with associations to enhanced tumor growth, immune modulation, and an increased potential for resistance and metastasis [15–17]. In this context, leveraging anti-nuclear antibodies with their natural affinity for nuclear material could transform exDNA into the ideal target, potentially broadening the application of ADCs in cancer treatment. Anti-nuclear antibodies are autoantibodies that recognize and bind to nuclear components, including DNA, histones, and various nuclear proteins. Such autoantibodies are well-known in clinical immunology as indicators of autoimmune diseases, particularly systemic lupus erythematosus, where they reflect an immune reaction against the body’s own nuclear material [18].

In this study, we present an innovative anti-DNA antibody-based technology that leverages nucleic acids in the tumor microenvironment for targeted drug delivery. We used a fully humanized version of the anti-DNA monoclonal antibody 3E10, named V66 [19], chemically linked to exatecan, a potent topoisomerase 1 (TOP1) inhibitor [20]. Originally isolated from a Lupus mouse model, 3E10 is notable among lupus autoantibodies for its benign toxicity and unique cell-entry mechanism that bypasses the conventional endocytosis and lysosomal degradation pathways that typically characterize ADCs, allowing cell penetration and nuclear accumulation [21]. The DNA-binding capability of 3E10 is critical for its ability to penetrate cells and nuclei [22], a process that relies on equilibrative nucleoside transporter 2 (ENT2) [21, 23–25], which is more abundantly expressed in cancer cells than in healthy tissues [26]. Versions of 3E10 have been used for specific tumor targeting in several prior studies to deliver fusion proteins, nanoparticles, and microtubule inhibitors for cancer treatment [27–29].

Importantly, our recent work has established that the tumor-targeting potential of antibodies in the 3E10 family is directly correlated with their DNA-binding affinities, as engineered antibodies with higher DNA binding affinities show increased accumulation in tumors [19]. Based on its high nucleic acid binding affinity and robust tumor targeting properties, we selected one variant, V66, as an advantageous platform for delivery of therapeutic cargoes to tumors. Here, we present V66-exatecan, an innovative and highly potent ADC that penetrates tumor cells, accumulates in their nuclei in vivo, and delivers effective doses of exatecan. This conjugate exploits the unique properties of V66, which binds to exDNA in the tumor microenvironment, facilitating targeted internalization via ENT2. Our comprehensive in vitro and in vivo studies demonstrate the remarkable efficacy of V66-exatecan. In vitro, V66-exatecan effectively induces tumor cell death across a broad range of cancer cell lines without the need for a specific antigen, while sparing non-tumoral healthy cells. In vivo, these results are further validated, showing significant anti-tumor effects in challenging cancer models, including a cell line-derived xenograft model of triple-negative breast cancer (TNBC) and a genetically engineered mouse model (GEMM) of medulloblastoma, where drug delivery is traditionally hindered by the blood-brain barrier (BBB). The most striking efficacy of V66-exatecan is observed in DNA damage repair (DDR)-deficient tumor models, where the constant basal level of DNA damage leads to the accumulation of exDNA, resulting in greater internalization of the ADC. Efficacy studies in these models revealed a high percentage of complete tumor regression. Extensive toxicity studies confirmed a substantial safety margin, with toxicity only manifesting at extremely high doses, further validating the selective targeting of V66-exatecan, which spares healthy tissues while effectively targeting tumor cells. This study positions V66 as a promising platform for targeted cytotoxic agent delivery, showing high specificity, efficacy, and potential to advance cancer therapy.

## Methods

### Cell culture

VC8 and VC8-BRCA2+, as previously described [30], PEO1 and PEO1-C4-2, gifted from T. Taniguchi [31], KPC (#153474, CancerTools), EMT6 (CRL-2755, ATCC), MIA PaCa-2 (CRL-1420, ATCC), HeLa (CRM-CCL-2, ATCC) U2OS (HTB-96, ATCC) and MC38 (CRL-2638, ATCC) cell line were cultured in DMEM supplemented with 10% fetal bovine serum (FBS) and 1% penicillin-streptomycin (Pen/Strep) at 37 °C, with 5% CO₂. HCT116 *IDH1*^+/+^, *IDH1* R132H/+ and SKOV3 (HTB-77, ATCC) were maintained in McCoy 5 A supplemented with 10% FBS and 1% Pen/Strep. DLD1 WT and DLD1 BRCA2KO cells were obtained from Horizon Discovery. DLD1 WT, DLD1 BRCA2KO, MDA-MB-231, K562 (CCL-243, ATCC) and A427 (HTB-53, ATCC) cells were cultured in RPMI supplemented with 10% FBS and 1% Pen/Strep at 37 °C, with 5% CO₂. B1-5 V and B2-4 V mouse cell lines derived from the spontaneous medulloblastoma model were provided by Z. Shen and cultured in Neurobasal supplemented with 10% FBS, 1% Pen/Strep and 1% L-Glut at 37 °C, with 5% CO₂. Cells were regularly tested for *Mycoplasma* (MycoAlert, Lonza). Mouse primary fibroblasts were isolated from the ears and tails of six-week-old female athymic J: Nu mice, as previously described [32], with minor Modifications. Briefly, tissue from three mice was harvested under sterile conditions, incubated in 70% ethanol for 5 min, and air-dried for an additional 5 min. Ears and tails were then minced into fragments (< 3 mm) and digested with Collagenase D for 90 min at 37 °C on a shaker. Digested tissue was transferred to a 10 cm dish containing 10 mL of complete medium (DMEM, 10% FBS, 1× Pen/Strep), placed in a 70 μm cell strainer, and mechanically dissociated using a syringe plunger for ~ 5 min, with occasional agitation. The cell suspension was collected in a 15 mL tube, and the strainer was rinsed with an additional 10 mL of medium to recover residual cells. Cells were centrifuged at 580 × g for 7 min at 4 °C, washed twice, and resuspended in complete medium supplemented with 250 ng/mL amphotericin B. Cultures were maintained at 37 °C in a 5% CO₂ humidified incubator.

### V66 antibody Preparation

The sequence of V66 and 3E10-D31N have been reported in Rackear et al. [19]. The chimeric and humanized antibodies were expressed in stably transfected CHO cells. The antibodies were purified from conditioned supernatant using a three-step process involving Protein-A affinity chromatography followed by anion exchange chromatography and, finally, cation exchange chromatography (Catalent, Madison, WI). The purified antibody was exchanged into PBS, pH 6.2 using tangential flow filtration (TFF).

### Synthesis of ADCs

ADCs were prepared using a cysteine conjugation approach (NJ Bio, Princeton NJ). Briefly, V66 monoclonal antibody (4.1 mg/mL in PBS pH 7.0 + 2 mmol/L EDTA) was treated with 12 molar equivalents of tris(2-carboxyethyl)phosphine (TCEP) for 2 h at 37 °C to achieve full reduction. Subsequently, 12 molar equivalents of the mc-VA-PAB linker–exatecan drug (from a 10 mmol/L dimethylacetamide (DMA) stock solution) were added to the fully reduced antibody, ensuring that the residual DMA concentration remained below 15% (v/v). The mixture was incubated at room temperature for 2 h, followed by purification using Tangential Flow Filtration (TFF) with a 10-kilodalton molecular weight cutoff (MWCO) to remove excess reagents. The resulting ADC buffer was exchanged for 10 mM histidine buffer, pH 5.4, and sterile filtered. Purity was assessed by size-exclusion chromatography, and the drug-to-antibody ratio was determined by LC-MS.

### Cell viability assays

All cell lines were seeded at a density of 500 cells per well in solid white 96-well plates (Costar) and incubated under the specified conditions in triplicate. After 24 h, the cells were treated with increasing concentrations of V66-exatecan, IgG1-exatecan (isotype control), or V66 antibody alone for 24 h. Following treatment, the media were replaced with fresh media, and the cells were allowed to grow for an additional 6 days. Cell viability was assessed on day 7 using the CellTiter-Glo Kit (Promega) according to the manufacturer’s protocol. Data are presented as means ± SEM. Mouse primary fibroblasts were seeded at a density of 2,000 cells per well and cultured under standard conditions until they reached confluence, as previously described [22]. At confluence, proliferation naturally ceased without signs of cell death. Viability at confluence was measured using the same CellTiter-Glo assay. Data are presented as means ± SEM.

### Co-treatment assays with cathepsin B and proteasome inhibitors

For cell viability assays, 2,000 MDA-MB-231 cells were seeded in 96-well plates and allowed to adhere overnight. The following day, cells were treated with V66-exatecan antibody-drug conjugate (ADC) at either a high (4 µM) or low (0.5 µM) concentration for 72 h, with or without prior inhibitor pre-treatment. Cells were pre-treated for 2 h with either the Cathepsin B (CatB) inhibitor CA-074Me (10 µM; Selleck Chemicals, #S7420) or the proteasome inhibitor MG132 (0.5 µM; InvivoGen, #tlrl-mg132) before ADC addition. Cell viability was assessed after 72 h using the CellTiter-Glo assay (Promega), according to the manufacturer’s instructions.

For immunoblot analysis, 250,000 MDA-MB-231 cells were seeded in 12-well plates and incubated for 24 h. Cells were then pre-treated with CA-074Me (10 µM) or MG132 (0.5 µM) for 2 h, followed by co-treatment with V66-exatecan (100 nM) for an additional 24 h. After treatment, cells were harvested and lysed for Western blot analysis as described below.

To assess the role of equilibrative nucleoside transporters (ENTs) in ADC internalization, 2,000 DLD1 cells were seeded in 96-well plates and incubated overnight. The following day, cells were treated with dipyridamole (25, 50, or 75 µM) for 24 h to block ENT activity. After pre-treatment, cells were co-treated with V66-exatecan ADC for 1 h, after which the media were replaced with fresh growth medium. Cell viability was assessed 72 h later using the CellTiter-Glo assay (Promega), according to the manufacturer’s instructions.

### Nuclease treatment

For nuclease treatment, cells were seeded at a density of 350,000 cells per well in 6-well plates. After 24 h, they were incubated at 37 °C for 30 min in PBS containing either DNase I (0.4 mg/mL; Stemcell Technologies, #100–0762), RNase I (100 U/mL; Thermo Fisher Scientific, # EN0601), or Benzonase (200 U/mL; Sigma-Aldrich, # E1014) to ensure optimal enzymatic activity. Without removing the nucleases, cells were then incubated with media containing the V66 antibody for 1 h, followed by total lysis for downstream applications.

For subcellular fractionation experiments, a milder DNase I treatment was applied. Cells were incubated with DNase I (0.2 mg/mL in PBS) for 10 min at 37 °C, washed once with PBS, treated with V66 antibody in fresh media for 1 h, and subsequently trypsinized and processed for fractionation.

### Cell fractionation and protein extraction

All cell lines were harvested via trypsinization (minimum 1 × 10^6^) to collect for pelleting. Pellets were washed once in 1 mL PBS (Gibco, Cat. # 14190250). The cellular fractionation was performed as previously described [33]. Briefly, 5 × 10^6^ cells were pelleted and washed with PBS; then they were resuspended in the appropriate amount of Buffer A (200 mM Tris HCl pH 8, 10 mM NaCl, 3 mM MgCl_2_, 0.1% NP40, 10% glycerol, 0.2 mM EDTA, 1 mM DTT) complemented with Protease Inhibitor Cocktail (PIC) 1× (Complete, EDTA free, Roche). Cells were incubated on ice in Buffer A for 15 min, and then centrifuged at 200xg for 5 min at 4 °C. The supernatant contained the cytoplasmic extract. Buffer A was added to the pellet containing the nuclei, and the resuspended pellet was centrifuged again for washing. After washing twice, the pellet was resuspended in the same amount of Buffer C (20 mM Tris HCl pH 8, 400 mM NaCl, 20% glycerol, 1 mM DTT) complemented with PIC 1×. The nuclei were subjected to thermal shock with 3 cycles of freezing in liquid nitrogen and thawing at 37 °C. After thermal shock the extract was centrifuged at max speed for 15 min at 4 °C. The supernatant contained the nuclear extract. For total protein extraction, cells were lysed in AZ lysis buffer (50 mM Tris, 250 mM NaCl, 1% Igepal, 0.1% SDS, 5 mM EDTA, 10 mM Na_2_P_2_O_7_, 10 mM NaF) supplemented with PIC 1× and PhosSTOP 1× (Sigma). For tumor studies, tumors were dissected and dissociated to single-cell suspension using a Human Tumor Dissociation Kit (Miltenyi Biotech, #130-095-929) and lysate using RIPA Buffer (Thermo Fisher, Cat. #89900) supplemented with PIC 1× and PhosSTOP 1× for total protein extract of Buffer-A/Buffer-C for cellular fractionation. Band intensities were quantified using Image Lab software.

### Western blotting and antibodies

Cell lysates were run on 4–20% gradient SDS-PAGE gels (Bio-Rad StainFree TGX) and transferred for western blotting. The following antibodies were used for protein detection: GAPDH (Protein Tech, HRP-60004), TOP1 (Abcam, ab85038), pATR (phospho-Thr1989) (GeneTex, GTX128145), pATM (phospho-Ser1981) (Cell Signaling Technology, 13050 S), γH2AX (Cell Signaling Technology, 2577 S), anti-FC (Invitrogen, 31413).

### RNA extraction and qRT-PCR analysis

Total RNA was extracted from DLD1 WT and DLD1 BRCA2KO cell lines using the QIAGEN RNeasy Kit according to the manufacturer’s instructions. RNA yield was quantified using a NanoDrop One spectrophotometer (Thermo Fisher Scientific). For mRNA analysis, reverse transcription to cDNA was performed with the High-Capacity cDNA Reverse Transcription Kit (Thermo Fisher Scientific, #4368814), following the manufacturer’s protocol. Quantitative RT-PCR was performed on an Applied Biosystems 7500 Fast Real-Time PCR System. Reactions were carried out in triplicate using the SYBR Green dye detection system and analyzed with the 7500 Software v2.0.6 (Applied Biosystems). Relative expression levels of target genes were determined using the comparative 2∆∆Ct method. ActB mRNA was used as the reference gene.

**ActB primers:**

hACTB_FW_ZI: CGAGAAGATGACCCAGATCA

hACTB_REV_ZI: GTACAGGGATAGCACAGCC

**ENT2 primers:**

hENT2_FW_ZI: TGTTGGTCTTCACAGTCACC

hENT2_REV_ZI: AAGAACTGACTCCACTTCCC

### Co-culture and flow cytometry analysis of HCT116 cells

HCT116-GFP-negative cells (1 × 10⁶) were seeded in 6 cm culture dishes and treated with either V66 antibody or V66-exatecan antibody-drug conjugate (ADC) at concentrations of 0.1 µM and 0.5 µM for 24 h. Following treatment, cells were trypsinized and re-plated in fresh medium (without treatment) into new 10 cm dishes, together with 5 × 10⁵ HCT116-GFP-positive cells. Co-cultures were incubated at 37 °C with 5% CO₂ for an additional 48 h. After incubation, cells were trypsinized and centrifuged at 300 x g for 5 min. Following a wash with 1X PBS, cells were centrifuged again and resuspended in 1% paraformaldehyde (PFA) in 1X PBS at room temperature for 15 min. Samples were centrifuged at 300 × g for 5 min, washed once with 1× phosphate-buffered saline (PBS), centrifuged again, resuspended in 500 µL of 1× PBS and diluted 1:2. The cell suspension was then filtered through a 0.35 μm cell strainer. Samples were analyzed for GFP fluorescence using a CytoFLEX LX Flow Cytometer (Beckman Coulter). All samples were analyzed at a constant flow rate and recorded for 180 s. Gating and quantification were performed using FlowJo software.

### Flow cytometry for cell penetration of V66 at 4 °C

K562 cells were pre-treated at 4◦C or 37◦C for 45 min, then treated with 1 µM 3E10 for 1 h. Cells were collected and fixed and permeabilized with True-Nuclear 1X Fix Concentrate (BioLegend) and True-Nuclear 1X Perm Buffer (BioLegend), respectively, according to manufacturer protocol. The primary antibody used was AlexaFluor 488-conjugated AffiniPure goat anti-human IgG Fc (Jackson Immuno #109-545-170). This antibody was diluted 1:100 in blocking buffer and incubated at room temperature for 90 min. Samples were centrifuged at 300 x g for 5 min, washed with 1X PBS, centrifuged again, resuspended in 300 µML 1X PBS, and passed through a 0.2 μm cell strainer. Samples were analyzed for Al-488 fluorescence using a CytoFLEX LX Flow Cytometer (Beckman Coulter). Gating and quantification were performed on FlowJo v10 (Becton Dickinson & Company).

### Flow cytometry for cell penetration of V66

V66 antibody was directly labeled with IVISense 680 NHS Fluorescent Dye (Revvity) in 50 mM carbonate/bicarbonate buffer, pH 8.5 for 2 h at room temperature and purified using Zeba spin desalting columns. MC38 or U2OS cells were pre-treated with inhibitors for 30 min, then treated with inhibitors and 750 nM Al680-labeled antibodies at 37◦C, 5% CO2 for 1 h. Following treatment, cells were collected and passed through a 0.2 μm cell strainer for flow cytometry. Chemical inhibitors were obtained from the following sources and used at the following concentrations: Chlorpromazine HCl (Cayman Chemical), 5 µg/mL; Filipin III (Cayman Chemical), 1 µg/mL; Dipyridamole (Sigma Aldrich), 50 µM; S-(4-Nitrobenzyl)−6-thioinosine (Millipore Sigma), 100 µM. Three biological replicates were performed, and statistical significance was determined using two-way ANOVA.

### Immunofluorescence

For internalization experiments, 50,000 DLD1 cells were seeded in Millicell EZ chamber slides (Millipore) pre-coated with Collagen I (final concentration: 20 µg/mL). For cell penetration assays, cells were pre-treated with 50 or 100 µM dipyridamole (Sigma-Aldrich) for 30 min at 37 °C, followed by incubation with 100 nM antibody for 1 h. For lysosomal accumulation assays, V66 was conjugated to Zenon pHrodo IgG dye (Invitrogen, #Z25611) according to the manufacturer’s protocol, and HeLa cells were treated with 1 µM of the conjugated antibody for 4, 6, or 24 h. After antibody incubation, all cells were washed once with PBS and fixed for 15 min at room temperature using a solution containing 3% paraformaldehyde (Santa Cruz Biotechnology), 0.5% Triton X-100, and 8% sucrose (MilliporeSigma) in PBS. Samples were then washed twice with PBS and incubated overnight at 4 °C in blocking buffer composed of 5% normal goat serum (Invitrogen), 0.5% Triton X-100, and 8% sucrose in PBS. The following day, cells were stained for 2 h at room temperature with Alexa Fluor 647-conjugated goat anti-human Fcγ fragment secondary antibody (1:400; Jackson Immuno, #109-605-008). Nuclear DNA was counterstained with a 1:1 mixture of DAPI (Sigma-Aldrich, D9542) and Hoechst (Sigma-Aldrich, B2261) for 15 min at room temperature, followed by three 5-minute PBS washes. After washing, chambers were removed, and coverslips were mounted using ProLong™ Glass Antifade Mountant (Invitrogen, P36980). Slides were sealed with nail polish 48 h later and stored at − 20 °C, protected from light, for long-term storage.

### Extracellular DNA (exDNA) quantification

For in vitro quantification of extracellular DNA, 0.5 × 10⁵ DLD1 WT or DLD1 BRCA2KO cells were seeded into each well of a full 12-well plate (one plate per cell line) and cultured for 4 days under standard conditions. Culture supernatants were then collected and centrifuged at 1,000 × g for 10 min to remove cellular debris. The resulting cleared supernatants were analyzed using a NanoDrop One spectrophotometer to quantify extracellular double-stranded DNA (dsDNA), single-stranded DNA (ssDNA), and RNA levels. For in vivo detection of extracellular nucleic acids in tumors, mice bearing DLD1 WT or DLD1 BRCA2^KO xenografts were injected intravenously with 2.5 µM SYTOX™ Deep Red Nucleic Acid Stain (Invitrogen, Cat. #S11380), a cell-impermeant dye that selectively stains nucleic acids in dead or membrane-compromised cells. Approximately 6 h post-injection, tumors were excised, bisected, and imaged ex vivo using the IVIS Spectrum Imaging System (PerkinElmer) to assess extracellular nucleic acid accumulation.

### Mouse tumor models

The Yale University Institutional Animal Care and Use Committee approved all mouse studies. In all cases, female mice 6 to 8 weeks of age were used and kept in temperature-controlled environments with 12-h light cycles and free access to water and food. Tumor cells were implanted as described for each model below, and mouse weights were recorded 3 times per week. In the case of flank tumor models, tumor volumes were calculated using calipers, and the following formula was used: V = 1/2(4π/3) (length/2) (width/2) (height). Mice were treated when tumors reached an average size of 100–200 mm^3^, and irradiation (10 Gy) was applied locally to tumors using an X-RAD 320 X-Ray Biological Irradiator (Precision X-Ray Inc).

For in vivo xenograft studies, 3 × 10^6^ human DLD1 BRCA2KO or 1 × 10^6^ human HCT116 cells in 100 µL of media were implanted subcutaneously in the flanks of 6–8-week-old athymic J: Nu female mice. For MDA-MB-231 xenografts, 5 × 10^6^ cells in 100 µL of media containing 50% Matrigel were implanted subcutaneously. For in vivo mouse tumor studies, 2 × 10^5^ mouse EMT6 cells in 100 µL of media were implanted subcutaneously in the flanks of BALBc/Rw mice. Spontaneous medulloblastoma mice (Brca1^−/−^; Trp53^−/−^ and Brca2^−/−^; Trp53^−/−^) were obtained as previously described [34].

### In vivo treatments and imaging

For efficacy and toxicity studies, mice were treated with V66 antibody, V66-exatecan antibody-drug conjugate, or exatecan mesylate (MedChemExpress, DX8951f) via intraperitoneal injection for 3 weeks, administered either 2 or 4 consecutive days per week, depending on the treatment group. For biodistribution studies, V66 and human IgG1 isotype control antibodies (Bio X Cell, Cat. #CP169) were conjugated to IVISense 680 NHS fluorescent dye (Revvity, Cat. #NEV11118) according to the manufacturer’s instructions. As an additional control, free fluorescent dye was included. Once tumors reached 150–200 mm³, mice were treated with 100 µg of the labeled antibodies via intravenous retro-orbital injection under 2.5% isoflurane anesthesia. Ex vivo fluorescence imaging was performed using the IVIS Spectrum In Vivo Imaging System (PerkinElmer). Mice were monitored post-injection, and tumors along with major organs were collected and imaged 24 h after injection to assess biodistribution. In a separate experiment, GEMM mice bearing spontaneous BRCA2-mutant medulloblastomas were injected with 100 µg of fluorescently labeled V66 antibody via retro-orbital intravenous injection. After 24 h, brains were harvested and imaged ex vivo using the IVIS Spectrum system to evaluate antibody accumulation in tumor-bearing tissue. Non–tumor-bearing control was also injected with 100 µg of labeled V66 and used to establish baseline background fluorescence levels in the brain. To assess extracellular nucleic acid content in tumors, Sytox Deep Red dye (Thermo Fisher Scientific, S11380) was prepared at a concentration of 2.5 µM and injected retro-orbitally according to the manufacturer’s protocol. Six hours post-injection, tumors were excised and subjected to ex vivo fluorescence imaging using the IVIS Spectrum system.

### Peripheral blood analysis

For peripheral blood collection, all mice were anesthetized with 2.5% isoflurane. Blood was collected retro-orbitally using heparinized micro-hematocrit capillary tubes (Fisher Scientific). For complete blood counts, 50 µL blood was collected into heparin-coated tubes containing 10 µL 0.5 mol/L EDTA acid, and analysis was performed using a Hemavet 950FS (Drew Scientific) according to the manufacturer’s protocol. For blood chemistry analysis, blood from vehicle- or V66-exatecan-treated mice was collected at the specified time points, and serum separated using lithium heparin and sent to Antech Diagnostics for analysis.

### Bone marrow

Bone marrow cells were harvested by flushing femurs and tibias from mice 48 h after intraperitoneal treatment with vehicle or V66-exatecan (5, 10, 25, 50, 100, 200 mg/kg). Cells were automatically counted using a Cellometer Auto T4 (Nexcelom).

### Pharmacokinetic ELISA analysis

A pharmacokinetic study of the monoclonal antibody V66 was conducted in naïve 7–8-week-old C57BL/6 mice. Serum samples were collected at baseline (0 hours), 5 minutes, 15 minutes, 30 minutes, and 1, 2, 4, 6, 12-, 24-, 72-, and 96-hours post-dose. Serum antibody concentrations were determined using an Fc capture ELISA on the MSD platform. Each sample was analyzed in triplicate, with results reported in µg/mL. The study was conducted at CRO, TD2. Briefly, MSD Multi-Assay 96-well plates (Cat# L15XA-3) were coated with goat anti-human Ig Fc (SouthernBiotech, Cat# 2047-01) at 2 µg/mL in PBS and incubated overnight at 4°C. Plates were blocked with 3% BSA in TBST for 1 hour, followed by the addition of standards and diluted serum samples, which were incubated at room temperature (RT) for 1 hour. After three washes with 1X TBST, plates were incubated with a SULFO-TAG (Cat# R31AA-1)-conjugated goat anti-human F(ab’)₂ antibody (Invitrogen, Cat# 31122; 2 mg/mL in 1% BSA/TBS) for 1 h at RT with shaking at 700 rpm. Following another three washes with 1X TBST, Read Buffer (Cat# R92TC-2) was added, and the plates were read on an MSD MESO QuickPlex SQ 120 instrument. Mean values of Ab concentration and the standard deviation of replicate results was evaluated for variability. If the standard deviation exceeded 30% of the average result, the sample was reanalyzed, and the repeat results were reported. This approach was also applied to assess inconsistencies within a time point that could not be attributed to sample collection or processing.

Pharmacokinetic parameters were analyzed using noncompartmental analysis in Phoenix WinNonLin. The following parameters were assessed and reported: half-life (t½), time to maximum concentration (Tmax), maximum concentration (Cmax), area under the concentration-time curve (AUC₀-last, AUC₀-inf, AUC % extrapolated), volume of distribution (Vz Obs), clearance (Cl Obs), and correlation coefficient (r²).

### Statistical analyses

Statistical analysis was performed in GraphPad Prism. Unless otherwise stated, data are presented as mean ± SEM. Comparisons between two groups were made with the two-tailed, unpaired t-test. ANOVA was used to compare multiple curves.

## Results

### Tumor-selective accumulation and nuclear localization of V66 antibody in vivo

Originally identified in a Lupus mouse model, the monoclonal anti-DNA antibody 3E10 exhibited a remarkable ability to selectively target tumors in vivo [19, 26–29]. Building on this foundation, we developed a fully humanized version of 3E10 antibody, designated V66, which has been previously demonstrated to target tumors with high precision [19]. The sequence of V66 contains multiple modifications in the complementarity determining regions (CDRs) relative to the parent 3E10, including a point mutation at position 31 of CDR1 in the heavy chain where asparagine replaces aspartic acid at position 31 (D31N). This results in higher DNA binding affinity and enhanced cellular penetration by expanding the positive charge within the putative DNA/RNA-binding pocket, thereby strengthening its interaction with nucleic acids [19, 26, 35]. The sequence of V66 and structural modeling of the expanded nucleic acid binding site relative to 3E10 has been reported [19].

**Fig. 1:**
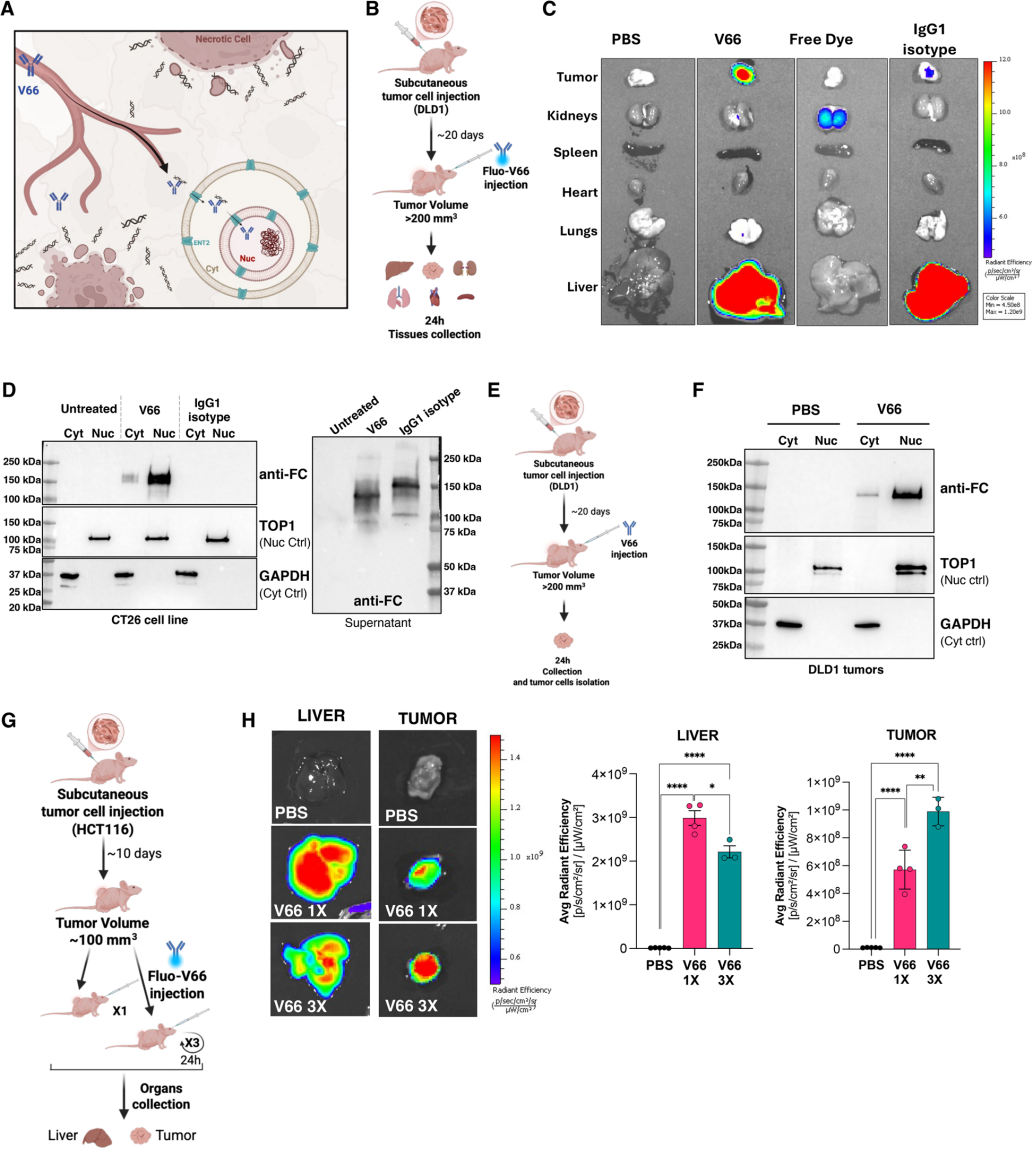
Systemically administered V66 antibody targets tumors, specifically distributing to malignant cells in vivo with high precision and penetrating their nuclei. **A** Cartoon representation of the mechanism of action of the V66 anti-DNA antibody after systemic administration, V66 targets extracellular DNA in the tumor microenvironment and is transported into tumor cell nuclei via ENT2 transporters. **B** Schematic of biodistribution experiment in athymic J: Nu mice with subcutaneous DLD1 tumors. Mice received 100 µg of dye-labeled V66 or IgG1 isotype control. Tissues were harvested 24 h post-treatment. **C** Representative IVIS Spectrum fluorescence images of tissues harvested from mice injected with V66, IgG1 isotype control, free dye or PBS as described in panel (B). **D** Western blot analysis was performed on fractionated CT26 cells following a 30-minute treatment with V66 or IgG1 isotype control antibodies, using a secondary antibody against the human Fc and normalized to specified nuclear (TOP1) and cytoplasmic (GAPDH) protein controls. Supernatant was also collected to detect the presence of excess antibodies. **E** Schematic of nuclear targeting experiment in athymic J: Nu mice with subcutaneous DLD1 tumors. Mice received 100 µg of V66 or PBS. Tumors were harvested 24 h post-treatment and cancer cells were isolated and fractionated in cytoplasmic and nuclear extracts. **F** Representative western blot analysis of fractionated tumor cells from mice injected with V66, as described in panel (**E**). A secondary antibody against human Fc was used to detect the V66 antibody. Protein levels were normalized to nuclear and cytoplasmic protein controls (*n* = 3). **G** Schematic of biodistribution experiment in athymic J: Nu mice with subcutaneous HCT116 tumors. One group received a single 100 µg dose of dye-labeled V66 (X1), and the other group received 100 µg doses of dye-labeled V66 daily for three consecutive days (X3). Tissues were harvested 24 h after the final treatment. **H** Representative IVIS images of collected tissues with relative quantification (data are presented as mean ± SEM, *n* = 3–4). (* *P* < 0.05, ** *P* < 0.01, *** *P* < 0.001, **** *P* < 0.0001, Student’s t-test, ns, not significant.)

The cartoon in Fig. 1A illustrates the mechanism of action of the V66 anti-DNA antibody. After systemic administration, V66 targets exDNA in the tumor microenvironment and enters tumor cell nuclei via the ENT2 nucleoside transporter, which is upregulated in multiple cancer types in patients compared to their matched healthy tissues (Fig. S1A), and is also elevated in mouse tumor cells relative to healthy tissues [26]. This process provides a dual-targeting mechanism, requiring both the presence of exDNA in the tumor microenvironment and ENT2 overexpression, making the antibody highly selective and minimizing off-target toxicity.

We conducted several experiments to evaluate antibody uptake and confirm its ability to penetrate cells. First, we established a tumor model by subcutaneously injecting DLD1 human colorectal cancer cells into the flanks of nude mice to assess the in vivo tumor-targeting efficiency of V66. When the tumors reached approximately 200 mm³ in size, the mice received an intravenous injection of either fluorescently labeled V66 antibody, fluorescently labeled human IgG1 isotype control antibody, free fluorescent dye, or vehicle (phosphate-buffered saline, PBS) (Fig. 1B). Twenty-four hours post-injection, the biodistribution of V66 was evaluated in tumor tissues as well as in a range of other major organs. The results revealed efficient and specific accumulation of V66 in the DLD1 tumors, with minimal signal detected in other tissues. Among non-malignant tissues, uptake was observed only in the liver, a finding consistent with previous studies showing that this uptake is associated with clearance by non-parenchymal cells, without involvement of hepatocytes [26]. In contrast, the fluorescently labeled IgG1 isotype control showed markedly lower tumor localization, consistent with nonspecific accumulation due to the enhanced permeability and retention (EPR) effect commonly observed in tumors. Free dye was primarily detected in the kidneys, reflecting its rapid clearance and lack of tumor-targeting capability (Fig. 1C).

To better understand the mechanism by which V66 enters tumor cells, we evaluated whether canonical endocytic pathways contribute to its internalization. We conducted a series of in vitro experiments designed to inhibit endocytosis. As an initial approach, we employed a commonly used method to broadly suppress endocytic processes: incubation at 4 °C, which inhibits energy-dependent uptake mechanisms, including clathrin- and caveolae-mediated endocytosis [36]. K562 cells were pre-incubated at either 37 °C–4 °C and subsequently treated with 1 µM of the humanized V66 antibody; as a positive control, cells were treated with chimeric 3E10-D31N, while buffer alone and human IgG1 isotype antibody served as negative controls. Flow cytometry analysis revealed no significant difference in uptake between the two temperature conditions (Fig. S1B), indicating that internalization occurs independently of conventional endocytic routes. We further examined the involvement of canonical endocytic pathways using small-molecule inhibitors. Cells were pre-treated with 5 µg/mL chlorpromazine, which blocks clathrin-mediated endocytosis, or 1 µg/mL filipin III, which disrupts caveolae-mediated uptake. After 30 min, V66 antibody was added at 1 µM in the continued presence of inhibitors. Immunofluorescence imaging in both murine (MC38) and human (U2OS) cell lines showed no significant change in antibody internalization (Fig. S1C). These results, consistent with the 4 °C inhibition assay, suggest that V66 penetrates cells through a non-endocytic mechanism. Lysosomal trafficking was also evaluated to determine whether V66 accumulates in acidic intracellular compartments. We employed a pH-sensitive dye conjugated to V66, which fluoresces only under the low pH conditions characteristic of lysosomes. HeLa cells were treated with 1 µM of the labeled antibody and monitored over time via immunofluorescence imaging. By 24 h, only about one-third of cells showed detectable green fluorescence, indicating limited lysosomal localization (Fig. S1D). These findings suggest that although a small proportion of internalized antibody may be entering the lysosomal pathway, this is not the predominant trafficking route for V66. Finally, to determine whether V66 utilizes the same transport mechanism described for the original 3E10 scFv [21, 22, 27, 37] or the chimeric versions 3E10 and 3E10-D31N [26], we evaluated the role of equilibrative nucleoside transporter 2 (ENT2) in antibody uptake. Cells were treated with dipyridamole, a broad-spectrum inhibitor of ENT1, ENT2, and ENT4, or S-(4-Nitrobenzyl)−6-thioinosine (NBMPR), which blocks ENT1 and ENT2 at higher concentrations [38]. In both murine (MC38) and human (U2OS) cell lines, treatment with either compound at 100 µM led to a pronounced reduction in nuclear accumulation of V66 (Fig. S1E). These results strongly support a model in which V66 relies on ENT2 activity for efficient cellular penetration and nuclear localization, reinforcing prior findings for the parental 3E10 antibody.

Tumor-targeting and biodistribution were further evaluated using EMT6 mouse breast cancer tumors implanted in syngeneic Balb/c mice (Fig. S2A), which demonstrated comparable tumor-specific accumulation of V66 in EMT6 tumors as in the DLD-1 tumors in Fig. 1C, underscoring the reproducibility of this mechanism across models.

In addition, we assessed the pharmacokinetics of V66, conducting a serum half-life study to evaluate its circulation time and clearance kinetics. Naïve C57BL/6 mice received a single intravenous dose of V66 at 25, 50, or 100 mg/kg, and blood samples were collected at 0.5, 15, and 30 min, as well as 1, 2, 4, 6, 12-, 24-, 72-, and 96-hours post-treatment. Serum antibody concentrations were measured using an ELISA-ECL assay on the MSD platform. The estimated half-lives of V66 were 40.5 h at 25 mg/kg, 49.4 h at 50 mg/kg, and 29.95 h at 100 mg/kg. (Fig. S2B). These half-lives are relatively short compared to those of the parent antibodies for most ADCs in clinical use, such as trastuzumab deruxtecan (Enhertu) which has a half-life of 5.7 days [39], providing for a potential safety advantage by reducing healthy tissue exposure.

We next validated the nuclear localization of V66 both in vitro and in vivo. Mouse colon cancer CT26 cells were treated for 30 min with 1 µM of V66 or an IgG1 isotype control. Western blot analysis to detect the antibody Fc domain revealed that V66 effectively penetrated the cells and predominantly localized in the nucleus, while the IgG1 control showed no evidence of cellular penetration (Fig. 1D). In these immunoblots, we used TOP1 and GAPDH as controls to validate the nuclear and cytoplasmic fractions, respectively. Additionally, analysis of the supernatant from both samples confirmed the abundant presence of antibodies in the media (Fig. 1D), demonstrating their availability for cellular uptake. Next, we sought to confirm the nuclear uptake of V66 in vivo. To this end, human colon cancer DLD1 cells were subcutaneously injected into the flanks of nude mice to form tumors. Once the tumors grew to a size of 200 mm³, the mice were administered 100 µg of V66 antibody (5 mg/kg) via intravenous injection (Fig. 1E). Tumors were harvested 24 h post-injection, and the tumor cells were dissociated, isolated, and fractionated into nuclear and cytoplasmic fractions. Western blot analysis of the fractions, as above to detect the antibody Fc domain, confirmed the predominantly nuclear localization of V66 in tumor cells in vivo (Fig. 1F).

To further investigate tumor versus liver localization and examine the role of the liver in antibody clearance, we compared antibody biodistribution following a single intravenous injection versus multiple injections of fluorescently labeled V66 in mice bearing HCT116 human colorectal subcutaneous tumors. Once the tumors reached approximately 100 mm³, mice were administered 100 µg of V66 either once or daily for three consecutive days. Tumors and livers were collected, and fluorescence was analyzed 24 h after the final injection. (Fig. 1G). The results showed that a single injection saturated the liver, with antibody signal decreasing over time, while the tumor continued to accumulate signal with each subsequent injection, in keeping with non-specific liver clearance and specific tumor targeting (Fig. 1H). Overall, these findings demonstrate the efficient targeting and selective accumulation of V66 in tumors, its effective nuclear localization, and its potential for safe and precise tumor-targeted therapy.

### Characterization of V66-exatecan ADC: nuclear penetration and impact on DDR activation

**Fig. 2:**
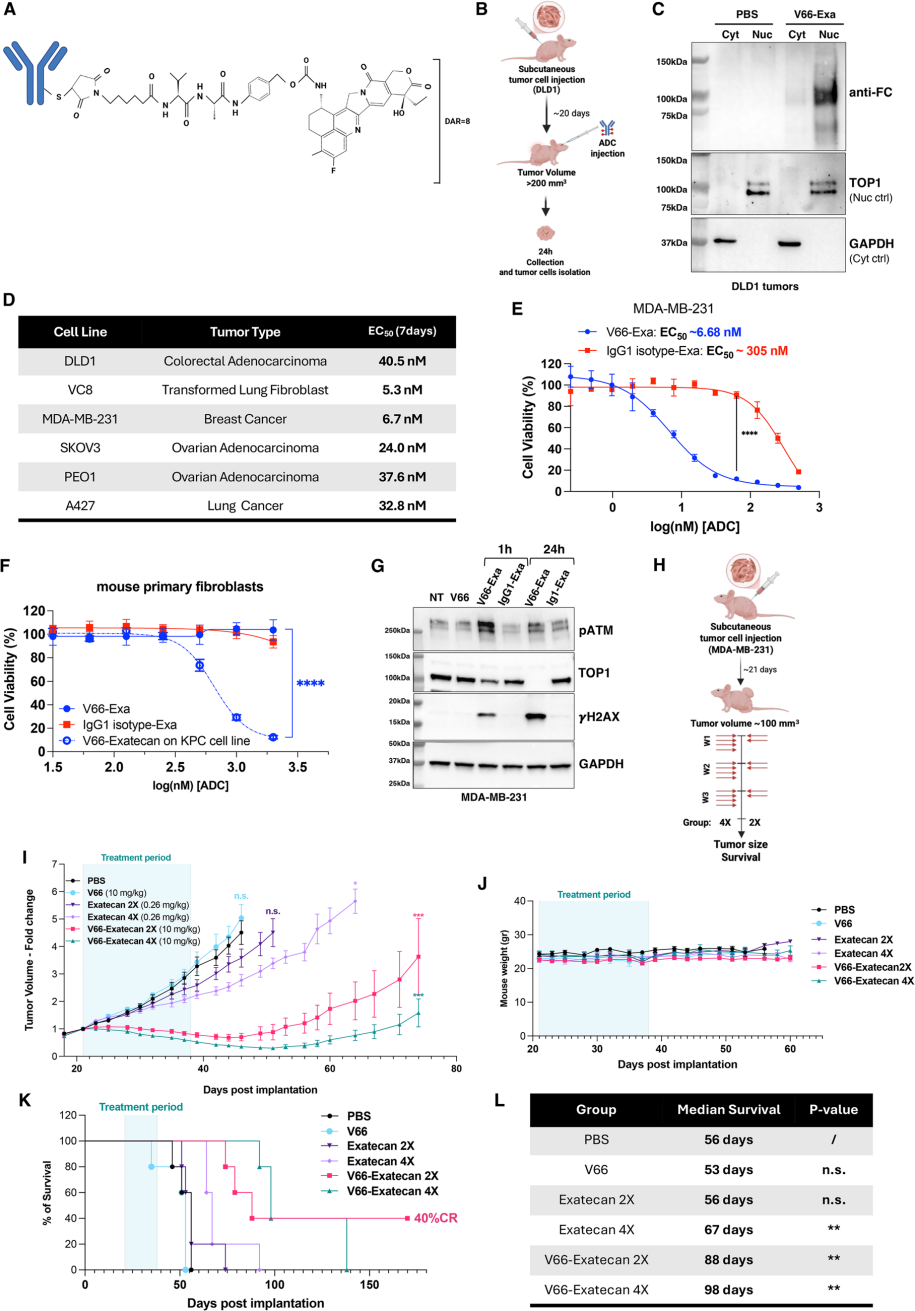
Development and characterization of V66-Exatecan ADC with potent nuclear targeting, DNA damage induction, and efficacy in Triple-Negative Breast Cancer. **A** Illustration of the V66 antibody structure chemically linked to the Exatecan drug via an mc-VA-PAB linker (DAR = 8). **B** Schematic of nuclear targeting experiment in athymic J: Nu mice with subcutaneous DLD1 tumors, administered 100 µg of V66-Exatecan or PBS. Tumors were harvested 24 h post-treatment and cancer cells were isolated and fractionated in cytoplasmic and nuclear extracts. **C** Representative western blot analysis of fractionated tumor cells, a secondary antibody against the human Fc was used to detect V66-Exatecan ADC, with normalization to nuclear and cytoplasmic protein controls (*n* = 3). **D** EC₅₀ values for various cancer cell lines treated with V66-Exatecan for 24 h. Cell viability was assessed 7 days post-treatment using the Cell Titer-Glo detection kit. **E** Dose-response curve comparing the cytotoxicity of V66-Exatecan (red) and IgG1 isotype-Exatecan (blue) in MDA-MB-231 cancer cell lines. Cells were treated for 24 h, and cell viability was assessed 7 days post-treatment using the Cell Titer-Glo detection kit. (Data are represented as mean ± SEM, *n* = 3). (****P < 0.0001, two-way ANOVA multiple comparison test). **F** Dose-response curve comparing the cytotoxicity of V66-Exatecan (red) and IgG1 isotype-Exatecan (blue) in mouse primary fibroblasts and murine KPC cell line. Cells were treated for 1 h, and cell viability was assessed 7 days post-treatment using the Cell Titer-Glo detection kit. (Data are represented as mean ± SEM, *n* = 3). **G** Western blot analysis of MDA-MB-231 cells treated with PBS, V66 (24 h), or V66-Exatecan ADC for 1–24 h. In samples treated with V66-Exatecan ADC, a time-dependent accumulation of DNA damage is observed, as indicated by the increased γH2AX signal, along with a time-dependent downregulation of the Exatecan target Topoisomerase 1. **H** Schematic of efficacy experiment in athymic J: Nu mice with subcutaneous MDA-MB-231 tumors: mice received 10 mg/kg of V66 or V66-Exatecan and equimolar dose of Exatecan Mesylate (0.26 mg/kg). Injections were performed 4 (4X) or 2 (2X) consecutive days a week for 3 weeks. **I** V66-Exatecan suppresses triple-negative breast cancer tumor growth in a dose-dependent manner. The graph shows tumor volumes in mice bearing MDA-MB-231 subcutaneous xenograft flank tumors (*n* = 5 per group). The treatment period is indicated by the light blue section. (*P < 0.05, ***P < 0.001, n.s. not significant, Two-way ANOVA multiple comparison test). **J** Body weight over time of mice in the study from panel (H). Treatments were well tolerated across all groups with different injection schedules. **K** Kaplan-Meier survival curves of mice in the study from panel (H) following treatment with V66-Exatecan or free drug. CR, complete regression. **L** Median survival for each group measured in days. Prolonged survival was observed in mice treated with the highest dose of free drug and all groups treated with V66-Exatecan. Statistical analysis was performed using the Log-rank (Mantel-Cox) test (**P < 0.01)

The unique tumor selectivity, efficient cellular penetration, and nuclear localization of V66 provide a strong rationale for its use in an innovative ADC approach. We conjugated V66 with exatecan, a synthetic derivative of camptothecin and potent inhibitor of nuclear TOP1 via an mc-VA-PAB linker attached to cysteine residues on the antibody (Fig. 2A). This linker is protease cleavable, specifically by cathepsin-related proteases. Cathepsins, including cathepsin B (CatB), are primarily located in endosomes but also found in the nucleus [40, 41], where the V66 antibody localizes after cell penetration. Exatecan as an ADC payload has shown promising efficacy in clinical trials but has faced dose-limiting toxicities as a monotherapy [20]. Nonetheless, ADCs with camptothecin derivatives, such as trastuzumab deruxtecan (Enhertu) for HER2-positive cancers and sacituzumab govitecan (Trodelvy) for Trop-2-expressing cancers, have demonstrated significant efficacy, underscoring the clinical potential of camptothecin-based payloads in oncology [42, 43].

To assess the quality of the V66 ADC, we conducted high-performance liquid chromatography (HPLC) analysis, which showed that the V66-exatecan conjugate exhibited nearly 100% purity (Fig. S3A). Additionally, mass spectrometry analysis demonstrated a drug-to-antibody ratio (DAR) of 8 (Fig. S3B). To validate that the ADC retains the cellular penetration capability of the parent antibody, we assessed in vitro nuclear localization and ENT2-dependent uptake of the V66-exatecan ADC. DLD1 cells were seeded onto collagen-coated slides and treated with 100 nM of either V66, V66-exatecan ADC, IgG1 isotype control, or PBS. In parallel, a subset of cells was pre-treated with 50 or 100 µM of dipyridamole to determine whether ADC internalization remains ENT2-dependent. Immunofluorescence analysis revealed efficient cellular uptake and predominant nuclear localization for both V66 and its ADC counterpart, while no internalization was observed for the IgG1 isotype control. Importantly, increasing concentrations of dipyridamole progressively reduced V66 and ADC uptake, confirming that the mechanism of internalization remains reliant on ENT2 (Fig. S3C).

To further confirm preservation of V66 biological activity in vivo, we assessed nuclear localization of the V66-exatecan ADC in tumor cells using the same approach described for the experiments in Fig. 1E–F. Mice bearing subcutaneous flank tumors of human DLD1 colon cancer cells received intravenous injections of 100 µg of the ADC once tumors reached approximately 200 mm³ (Fig. 2B). Tumors were collected 24 h post-injection, and cells were dissociated and fractionated. Western blot analysis of the nuclear and cytoplasmic fractions was conducted to evaluate the tumor cell penetration and subcellular localization of the ADC. The results confirmed that the conjugation of exatecan to V66 did not alter the biological properties of the V66 antibody, as the V66-exatecan ADC penetrated tumor cells and predominantly localized to the nucleus (Fig. 2C). These results indicate successful delivery of the ADC to the tumor cell nuclei, both in vitro and in vivo.

Next, we assessed the tumor cell killing efficacy of V66-exatecan in a panel of diverse cancer cell lines using the CellTiter-Glo viability assay. Cells were treated with increasing concentrations of the V66-exatecan ADC for 24 h, and their viability was assessed after 7 days. Treatment with V66-exatecan showed potent anti-tumor cell activity, with low nanomolar EC_50_ values across various tumor cell types (Fig. 2D). Nonetheless, there was some variation in EC_50_ values, and so to test for a correlation of the EC_50_ values with ENT2 expression, we performed RT-PCR analyses for ENT2 expression in the cancer cell lines (Fig. S3D). While all the lines expressed substantial ENT2, we did observe some cell line to cell line variability, but this variability did not directly correlate with the sensitivity to the ADC. However, the multiple assays above (Figs. 1D, F and G and 2 C) for antibody uptake across different cancer cell lines (DLD-1, DLD-1 BRCA2, HCT116, MDA-MB-231, and CT26) consistently show robust cell penetration and nuclear localization of the antibody in all the cancer lines tested, suggesting that there is sufficient ENT2 expression in all these tumor cell lines to support strong antibody penetration. Based on this, we hypothesize that differences in sensitivity of the cancer cell lines tested may reflect differences in cell background and intrinsic sensitivity to the topoisomerase I inhibitor payload more than differences in ADC uptake.

Next, to further interrogate whether the cytotoxic effect of the ADC relies on ENT2-mediated uptake, DLD1 cells were pre-treated for 24 h with increasing concentrations of dipyridamole (25, 50, or 75 µM), followed by a 1-hour exposure to 0.5 µM V66-exatecan ADC. Cell viability was then measured after 72 h. As expected, dipyridamole pre-treatment reduced the cytotoxic activity of the ADC in a dose-dependent manner, further supporting the importance of ENT2 in mediating ADC internalization and subsequent drug delivery (Fig. S3E).

To investigate the bystander killing potential of V66-exatecan, we performed a co-culture assay using HCT116 parental cells (GFP-negative) and HCT116-GFP-positive cells. Parental HCT116 cells were treated with 0.1 or 0.5 µM of either unconjugated antibody or V66-exatecan ADC for 24 h. Following treatment, cells were co-cultured with an equal number of untreated HCT116-GFP-positive cells. GFP-positive and GFP-negative populations were quantified by flow cytometry 48 h after co-culture. A marked reduction in both GFP-negative and GFP-positive cells was observed in the ADC-treated conditions compared to the antibody control or untreated cells, indicating a significant bystander killing effect mediated by V66-exatecan (Fig. S3F).

To further characterize the mechanism of action of V66-exatecan, we aimed to examine the cleavage of the linker and the release of the payload, as well as its impact on the cellular processes involved. Our results above, together with previous data from our lab [19], suggest that the V66 antibody enters cells through a unique mechanism that bypasses the lysosomal pathway. However, some reports have localized CatB not only in the lysosomal compartment but also in the nucleus of certain cancer cell lines [41]. We assessed cell viability in MDA-MB-231 cells after 72 h of treatment with high (4 µM) and low (0.5 µM) concentrations of V66-exatecan ADC, both with and without the presence of the CatB inhibitor CA-074Me. Co-treatment with CA-074Me (10 µM) partially rescued cell viability, indicating that Cathepsin B-mediated degradation may contribute to the cytotoxic effects of V66-exatecan (Fig. S3G). Additionally, we performed Western blot analysis to monitor key DDR markers, including pATM and γH2AX, as well as the degradation of the target protein TOP1. Co-treatment with CA-074Me partially modulated these effects, further supporting the involvement of CatB in the release of Exatecan. Complete rescue of TOP1 levels and full inactivation of DDR occurred only at a very high, and cytotoxic, doses (Fig. S3H). Next, we investigated the role of proteasomal degradation in the release of Exatecan. The intracellular Fc portion of the antibody is recognized by TRIM21, leading to antibody degradation [44]. Surprisingly, co-treatment with the proteasome inhibitor MG132 (0.5 µM) resulted in increased mortality of MDA-MB-231 cells after 72 h of treatment with either 0.5 µM or 4 µM V66-exatecan, likely due to the accumulation of stable TOP1-DNA complex inactivated by exatecan (Fig. S3I). In parallel, we performed Western blot analysis to examine DDR markers and TOP1. DDR activation decreased as MG132 concentration increased, with partial rescue of TOP1 protein observed starting at 0.1 µM MG132 (Fig. S3J). However, MG132 at higher concentrations (2.5-5 µM) was highly toxic to the cells. Together, these findings highlight the promise of V66-exatecan as a potent and selective therapeutic agent, capable of delivering a cytotoxic payload to the nucleus in a wide range of tumor types.

### Therapeutic efficacy of V66-exatecan ADC in TNBC with tumor regression and improved survival

Among the diverse panel of cell lines tested, the TNBC cell line MDA-MB-231 stood out as one of the most sensitive human cell lines, displaying an EC_50_ of 6.7 nM (Fig. 2D). This sensitivity highlights the potential of V66-exatecan as a promising therapeutic strategy for addressing the challenges associated with treating this aggressive cancer subtype. In Fig. 2E we show a representative plot of the cell viability assay, where V66-exatecan demonstrated significantly higher potency compared to an IgG1 isotype control-exatecan ADC, with an almost 50-fold difference EC_50_. In addition, mouse primary fibroblasts derived from ear skin showed no signs of toxicity when treated with either V66-exatecan or the IgG1 isotype control–exatecan ADC, in contrast to the mouse pancreatic cancer cell line KPC, which exhibited significant sensitivity to V66-exatecan under the same conditions (Fig. 2F).

Next, we used western blotting to further demonstrate the specificity of the effect observed in the viability assay for V66-exatecan versus the isotype control ADCs by assaying for induction of DDR signaling in the form of phosphorylated ATM (pATM) and γH2AX in the treated cells. MDA-MB-231 cells were treated with 100 nM of V66-exatecan or IgG1 isotype control for 1–24 h and analyzed for DDR induction. The results revealed a significant activation of the DDR pathway, as evidenced by the elevated signals of γH2AX and pATM which were specific to samples treated with V66-exatecan but not the isotype control ADC. There was also a time-dependent down-regulation of TOP1 expression in samples treated with V66-exatecan, but not in those treated with the isotype control-exatecan ADC (Fig. 2G). The downregulation of TOP1 in V66-exatecan-treated samples indicates successful TOP1 target engagement and inhibition, leading to degradation, a known effect of camptothecin-related TOP1 inhibitors [45]. As an additional comparison, the V66 antibody itself had no significant effects on TOP1 levels or DNA damage markers in the absence of the exatecan payload.

The activity of V66-exatecan was further assessed in vivo by assaying for anti-tumor efficacy using a set of four different mouse tumor models: two subcutaneous xenograft tumors models and two autochthonous brain tumor models. First, mice bearing subcutaneous MDA-MB-231 TNBC tumors were randomized to receive intraperitoneal injections, two (2X) or four times (4X) weekly, of either control buffer (PBS), V66-exatecan (10 mg/kg), unconjugated exatecan mesylate (equimolar dose, 0.26 mg/kg), or V66 antibody alone (10 mg/kg) (*n* = 5 mice per group) (Fig. 2H). The primary study endpoints included tumor volume, survival, and changes in body weight and behavior. V66-exatecan demonstrated a dose-dependent suppression of tumor growth compared to both the control buffer and exatecan mesylate (*P < 0.05, ***P < 0.001, two-way ANOVA multiple comparisons test) (Fig. 2I). Additionally, the V66 antibody alone showed no therapeutic efficacy when used independently of a conjugate. All doses were well tolerated, as no change in behavior or mouse weight was detected (Fig. 2J). Notably, V66-exatecan treatment significantly prolonged survival when compared to either the buffer control, exatecan mesylate, or unconjugated V66, highlighting the specific therapeutic efficacy of the ADC (Fig. 2K). Free exatecan mesylate administered four times a week did increase the median survival from 56 days to 67 days (Fig. 2L, **P < 0.01, log-rank (Mantel-Cox) test). However, V66-exatecan treatment, administered twice a week or four times a week, resulted in even greater survival benefits, extending the median survival to 88 and 98 days, respectively (a ~ 2-fold increase). Taken together, these findings demonstrate that systemic administration of V66-exatecan ADC via intraperitoneal injections effectively suppresses tumor growth in a challenging breast cancer model, significantly enhancing survival while maintaining high tolerability with multiple doses. This suggests that V66-exatecan ADC could represent a promising therapeutic approach for difficult-to-treat cancers.

### V66-exatecan ADC demonstrates enhanced activity in BRCA2-deficient tumors

**Fig. 3:**
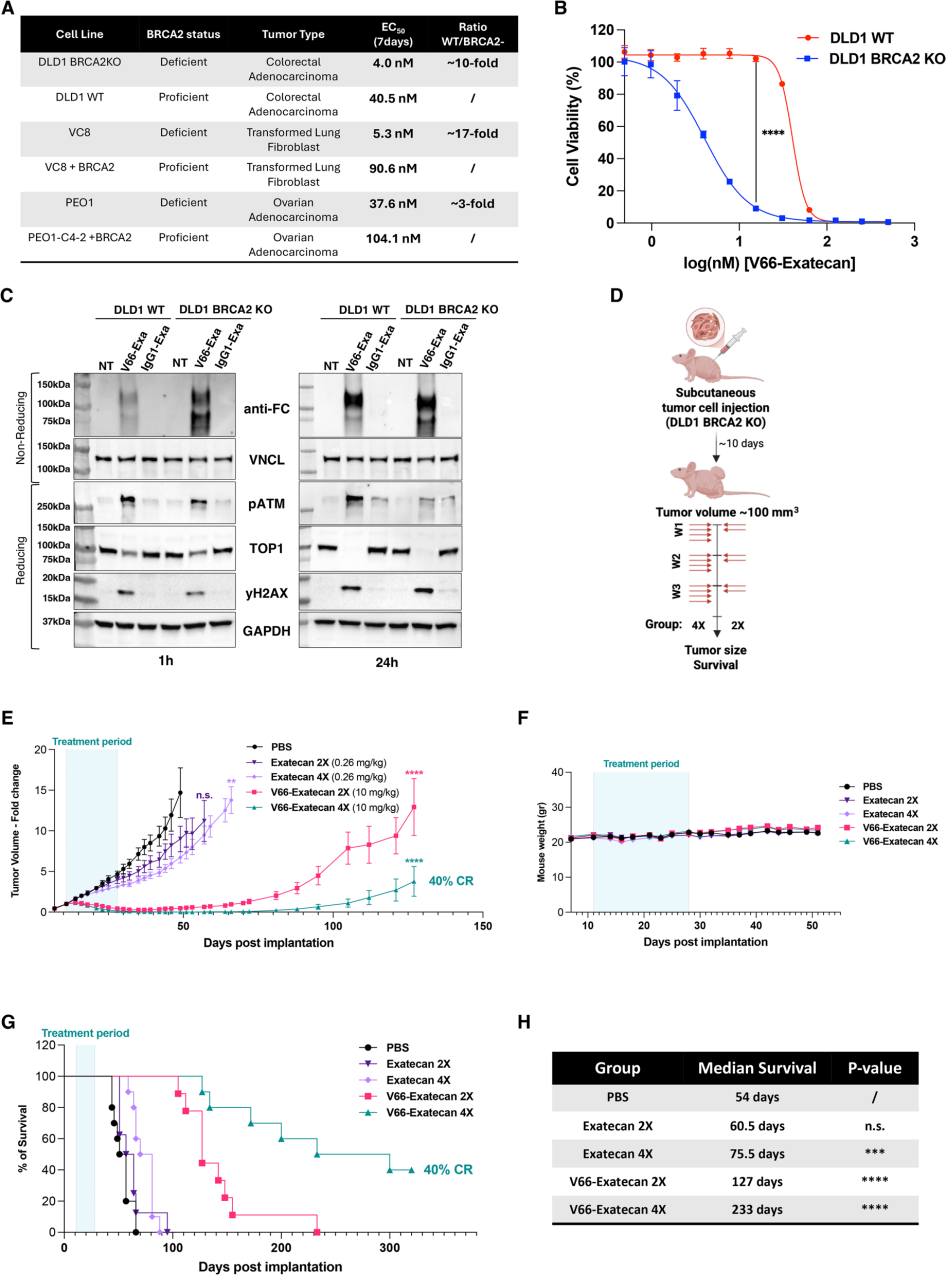
Enhanced sensitivity of BRCA2-deficient cell lines to V66-exatecan ADC, demonstrating greater therapeutic efficacy. **A** Comparison of EC50 values between BRCA2-proficient and BRCA2-deficient cancer cell lines treated with V66-exatecan for 24 h. Cell viability was assessed 7 days post-treatment using the CellTiter-Glo detection kit. The EC50 ratio of BRCA2-deficient to BRCA2-proficient cell lines is shown in the BRCA2-deficient group. **B** Dose-response curve comparing the cytotoxicity of V66-exatecan in DLD1 WT (red) and BRCA2KO (blue) cell lines. Cells were treated for 24 h, and cell viability was assessed 7 days post-treatment using the CellTiter-Glo detection kit. Error bars represent the standard deviation of triplicate measurements (data are presented as mean ± SEM, *n* = 3) (****P < 0.0001, two-way ANOVA multiple comparison test). **C** Western blot analysis comparing DLD1 WT and BRCA2KO cells treated with PBS, V66-exatecan, or IgG1 isotype-exatecan for 1–24 h. Upper panels: Non-reducing conditions reveal time-dependent accumulation of the V66 antibody, but not the IgG1 isotype control, with enhanced penetration observed in BRCA2KO cells compared to WT. Lower panels: Under reducing conditions, a time-dependent increase in DNA damage is indicated by elevated γH2AX and pATM levels, along with a progressive down-regulation of TOP1. **D** Schematic of efficacy experiment in athymic J: Nu mice with subcutaneous DLD1 BRCA2KO tumors. Mice received 10 mg/kg of V66-exatecan or an equimolar dose of Exatecan mesylate (0.26 mg/kg). Injections were performed 4 (4X) or 2 (2X) consecutive days a week for 3 weeks. **E** V66-exatecan suppresses BRCA2-deficient tumor growth in a dose-dependent manner. The plot illustrates the fold change in tumor volume (mean ± SEM) in mice bearing DLD1 BRCA2KO subcutaneous xenograft flank tumors (*n* = 10 per group). The treatment period is indicated by the light blue section (**P < 0.01, ****P < 0.0001, n.s., not significant, two-way ANOVA multiple comparison test). CR, complete regression. **F** Body weight over time of mice in the study from panel (E). Treatments were well-tolerated across all groups with different injection schedules. **G** Kaplan-Meier survival curves of mice in the study from panel (E) following treatment with V66-exatecan or free drug. CR, complete regression. **H** Median survival for each group measured in days. Prolonged survival was observed in mice treated with the highest dose of free drug and all groups treated with V66-exatecan. Statistical analysis was performed using the log-rank (Mantel-Cox) test (***P < 0.001, ****P < 0.0001, ns, not significant)

Around 10–15% of breast cancer cases in young women are diagnosed in patients with germline pathogenic or likely pathogenic variants in the BRCA1 or BRCA2 genes [46]. The *BRCA1* and *BRCA2* genes are tumor suppressors involved in DNA repair through homologous recombination, essential for maintaining chromosome integrity as part of the ATM-mediated DNA repair pathway [47]. Given their pivotal role in DNA repair, BRCA1/2 mutations can render tumor cells more susceptible to DNA-damaging agents like exatecan and other TOP1 inhibitors [48, 49]. To evaluate this, we tested a panel of matched cell lines with varying BRCA2 status using a viability assay. These included the DLD1 human colon cancer line with or without BRCA2 knockout (BRCA2KO or WT), as well as BRCA2-deficient cell lines, such as VC8 (Chinese hamster transformed lung fibroblasts) and Peo1 (human ovarian carcinoma), paired with their respective counterparts in which BRCA2 expression was restored. Cells were treated with increasing concentrations of the V66-exatecan ADC for 24 h, and their viability was assessed after 7 days. Sensitivity to V66-exatecan was drastically increased in all BRCA2-mutant cell lines, with a 10-fold increase observed for DLD1, a 17-fold increase for VC8, and a 3-fold increase for PEO1 (Fig. 3A). For the matched DLD1 cell lines, a representative survival curve after 7 days is shown in Fig. 3B, demonstrating a statistically significant difference in sensitivity between the BRCA2-proficient and BRCA2-deficient cells (****P < 0.0001, two-way ANOVA multiple comparison test).

We next treated DLD1 wild-type (WT) and DLD1 BRCA2KO cell lines for 1–24 h with 100 nM V66-exatecan to compare uptake, TOP1 target engagement, and induction of the DDR pathway with an IgG1 isotype-exatecan ADC or PBS buffer control (Fig. 3C). Our results confirmed the time-dependent down-regulation of TOP1 following V66-exatecan treatment, but not with the IgG1 isotype-exatecan ADC, and the concomitant activation of the DDR mechanism consistent with TOP1 inhibition. Only the V66-exatecan ADC, and not the IgG1-isotype-exatecan ADC, showed internalization as assayed by immunoblot under non-reducing conditions for the Fc domain. Notably, western blot analysis also suggested that there was greater internalization of V66-exatecan in the BRCA2-deficient cells compared to WT cells, a difference that is already evident at 1 h of treatment. (The basis for this difference in uptake will be explored further below).

The efficacy of V66-exatecan was next investigated in vivo using mice bearing subcutaneous DLD1 BRCA2KO tumors. Mice were randomized into groups (*n* = 10 per group) to receive either buffer control, V66-exatecan (10 mg/kg), or exatecan mesylate (equimolar dose of 0.26 mg/kg) via intraperitoneal injections administered two or four times weekly (Fig. 3D). The study assessed tumor volume, survival rates, and changes in body weight and behavior. V66-exatecan demonstrated dose-dependent tumor growth inhibition and induced pronounced tumor regression, showing significantly greater efficacy compared to exatecan mesylate (Fig. 3E). Notably, treatment with either regimen of V66-exatecan (2X and 4X weekly) resulted in multiple mice showing no visible tumors by the end of the treatment, with complete tumor regression observed in 40% of the mice in the V66-exatecan 4X group. All doses were well tolerated, as no change in behavior or mouse weight was detected (Fig. 3F). Mice treated with V66-exatecan demonstrated a striking improvement in survival compared to those receiving the control buffer or exatecan mesylate (Fig. 3G). While the exatecan mesylate administered four times a week modestly increased median survival from 54 days to approximately 76 days, V66-exatecan treatment, administered either twice or four times a week, provided even greater survival benefits, extending median survival to 127 days (~ 2.3-fold increase) and 233 days (~ 4-fold increase), respectively (Fig. 3H). Collectively, these data demonstrate that BRCA2 deficiency significantly increases tumor sensitivity to V66-exatecan, promoting tumor regression and significantly improving survival, highlighting the potential of V66-exatecan as an effective therapeutic strategy for BRCA2-deficient cancers.

### Extracellular DNA enhances V66 antibody uptake in DDR-deficient cells

**Fig. 4:**
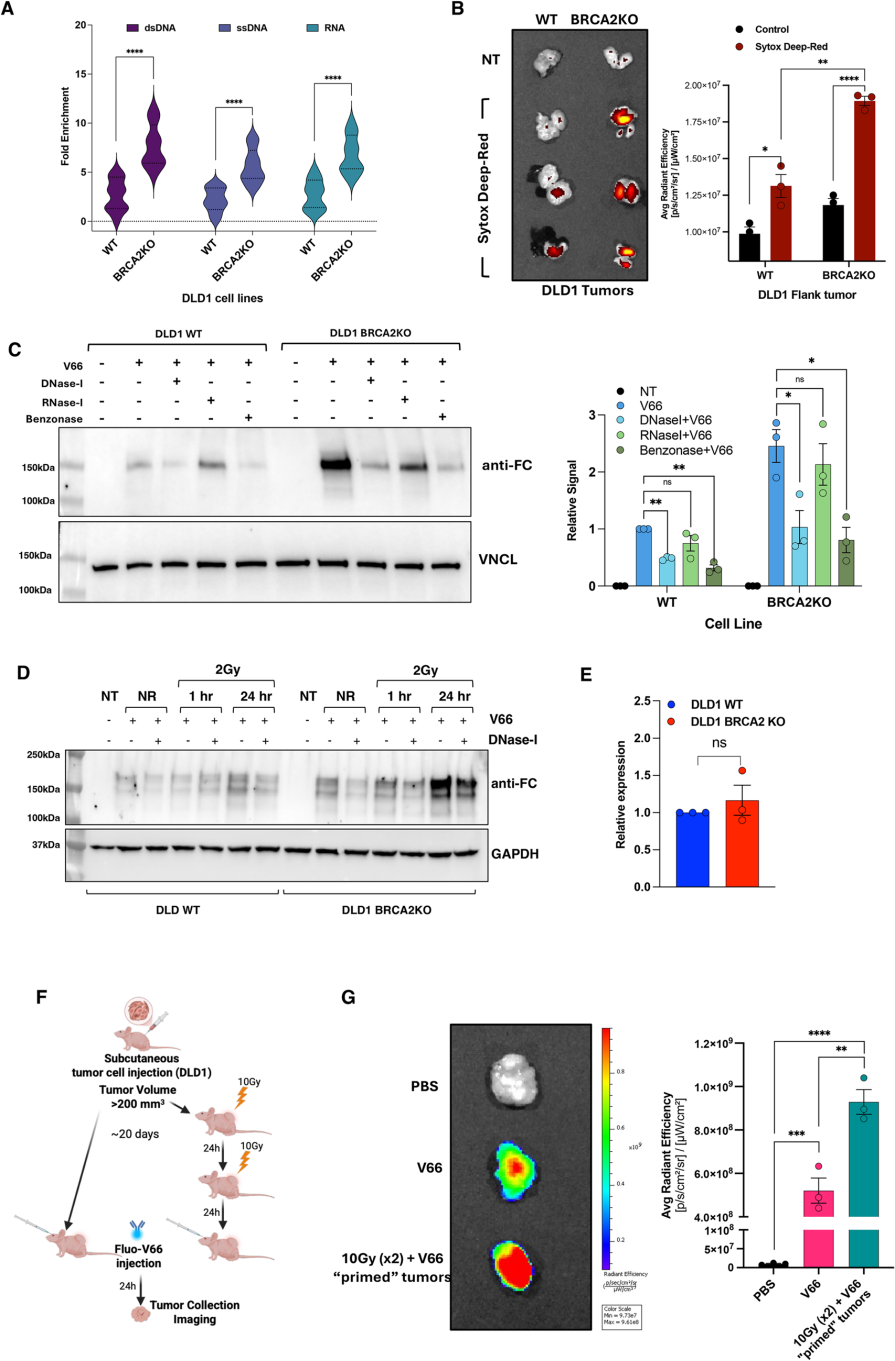
Enhanced penetration of V66 antibody in DDR-deficient cells and its dependence on exDNA. **A** Violin plot showing the levels of extracellular double-stranded DNA (dsDNA), single-stranded DNA (ssDNA), and RNA in the supernatant of DLD1 wild-type (WT) and BRCA2 knockout (BRCA2KO) cells. Cells were cultured for 4 days before collection of supernatants and quantification of exDNA and RNA. Each condition includes 12 technical replicates per biological replicate (*n* = 3). (**** *P* < 0.0001, Student’s t-test). **B** DLD1 WT (left) and BRCA2KO (right) tumors were excised from the flanks of nude mice 6 h after intravenous injection of Sytox Deep Red, a cell-impermeable dye that binds nucleic acids and selectively stains extracellular DNA or DNA from cells with compromised membrane integrity. Fluorescence imaging was performed to assess extracellular nucleic acid distribution within the tumor tissue. Relative quantification is shown in the right panel (*n* = 3) (* *P* < 0.05, ** *P* < 0.01, **** *P* < 0.0001, Student’s t-test). **C** Representative western blot analysis showing that exDNA influences V66 antibody internalization. BRCA2-deficient cells (KO) show greater internalization of V66 compared to DDR-proficient (WT) cells under normal conditions; however, pre-treatment with DNase-I or Benzonase (but not RNase-I) significantly reduces V66 penetration in both WT and BRCA2-deficient cells. Cells were pre-treated with DNase-I, RNase-I or Benzonase for 30 min, followed by 1 h of V66 treatment. Relative quantification is shown in the right panel. Data are presented as mean ± SEM (*n* = 3). (* *P* < 0.05, ** *P* < 0.01, Student’s t-test, ns, not significant). **D** Representative western blot showing increased V66 antibody internalization following radiation-induced DNA damage. Both WT and BRCA2KO cells exhibit enhanced internalization, particularly at 24 h post-irradiation, highlighting the dependence of penetration on the extent of DNA damage. Pre-treatment with DNase-I after radiation reduces internalization in both cell types, further confirming the role of extracellular DNA in facilitating V66 penetration (*n* = 3). NT, no treatment; NR, no radiation. **E** qRT-PCR analysis of ENT2 transporter expression in DLD1 WT and DLD1 BRCA2KO cell lines (*n* = 3). ENT2 mRNA expression levels were normalized to ActB as housekeeping gene. No significant differences in ENT2 expression were observed between DLD1 WT and BRCA2KO cells. **F** Schematic of biodistribution experiment in athymic J: Nu mice with subcutaneous DLD1 tumors. One group received a single 100 µg dose of dye-labeled V66, while the other group underwent “priming” with two 10 Gy radiation treatments, administered 24 h apart, to induce DNA damage before receiving 100 µg of dye-labeled V66. Tissues were harvested 24 h after the final treatment. **G** Representative IVIS images of collected tumors with relative quantification (data are presented as mean ± SEM, *n* = 3) (** *P* < 0.01, *** *P* < 0.001 **** *P* < 0.0001, Student’s t-test)

We hypothesized that our observation of increased V66 antibody internalization in BRCA2KO cells compared to WT cells (Fig. 3C) might reflect more exDNA accumulated in the media due to the reduced genome integrity and consequent increased basal level of DNA damage in the DNA-repair-defective BRCA2 deficient cells. We reasoned that this increased DNA damage could lead to elevated extracellular DNA which could enhance antibody internalization by increasing the interaction between V66 antibody and ENT2 transporters. Our prior work revealed enhanced penetration of the original 3E10 antibody in cell monolayers supplemented with extracellular DNA, supporting this hypothesis [22]. We first tested our hypothesis that BRCA2-deficient cells accumulate higher levels of extracellular nucleic acids via both in vitro and in vivo analyses. In vitro, equal numbers of DLD1 WT and BRCA2KO cells were cultured under identical conditions for four days. Conditioned media were then collected, cleared of debris and dead cells, and analyzed using NanoDrop spectrophotometry to quantify double-stranded DNA (dsDNA), single-stranded DNA (ssDNA), and RNA. BRCA2KO cultures exhibited a marked increase in extracellular nucleic acid species compared to WT counterparts (Fig. 4A). In parallel, in vivo studies were performed using subcutaneous xenografts of DLD1 WT and BRCA2KO cells in athymic nude mice. Once tumors reached approximately 200 mm^3^ in volume, mice were administered Sytox Deep-Red dye via retro-orbital injection (2.5µM). This cell-impermeable nucleic acid dye selectively stains exDNA or DNA from cells with compromised membrane integrity. Tumors were excised six hours post-injection and subjected to fluorescence imaging using an IVIS system. Consistent with the in vitro findings, BRCA2KO tumors showed significantly higher Sytox signal intensity, indicative of increased exDNA accumulation in vivo (Fig. 4B).

To further investigate the role of extracellular DNA (exDNA) in V66 internalization, we compared antibody uptake in DLD1 WT and BRCA2KO cell under basal conditions and following enzymatic degradation of nucleic acids using DNase I, RNase I, or Benzonase, targeting DNA, RNA, or both, respectively. Western blot analysis of the V66 Fc domain in nuclear and cytoplasmic fractions confirmed enhanced antibody uptake in BRCA2KO cells relative to WT, consistent with prior observations. Notably, treatment with DNase I or Benzonase markedly reduced V66 internalization in both cell types, supporting a role for extracellular DNA in mediating uptake. In contrast, RNase I treatment had minimal effect on V66 localization (Fig. 4C).

To test whether these findings could be seen in other cell lines with distinct mutations in the DDR pathway, we repeated the experiment in a matched pair of WT and IDH1 mutant cell lines in the HCT116 human colon cancer background. IDH1 mutations disrupt cellular metabolism by generating abnormally high levels of the oncometabolite 2-hydroxyglutarate causing a defect in the homologous recombination pathway at the level of chromatin signaling and DNA repair factor recruitment, leading to increased DNA damage and a compromised DDR [50]. The HCT116 IDH1-mutant cells showed increased accumulation of the V66 antibody under normal conditions compared to the parental line, aligning with observations in BRCA2-deficient cells. DNase-I treatment of the media substantially reduced the elevated internalization in both HCT116 WT and IDH1 mutant cells (Fig. S4A).

To further investigate the relationship between DNA damage and V66 antibody internalization, we designed an experiment to increase DNA damage levels in the cells. Both WT and BRCA2-deficient DLD1 cell lines were exposed to 2 Gy of radiation, either 1–24 h before V66 treatment. Western blot analysis revealed a time-dependent increase in V66 internalization following radiation exposure, observed in both the DLD1 WT cells and DLD1 BRCA2KO cells (Fig. 4D). These increases in antibody uptake were reduced by DNase-I addition to the media. To exclude the potential role of the ENT2 transporter in the observed differences in V66 antibody internalization, we performed quantitative real-time PCR (qRT-PCR) to measure ENT2 expression levels in both DLD1 WT and BRCA2KO cell lines. The qRT-PCR was performed in triplicate for each cell line, and no significant differences in ENT2 mRNA expression were observed between the two cell lines (Fig. 4E). These findings were further corroborated by immunofluorescence analysis in Mia-PaCa2 cells, where treatment with V66 pre-complexed with double-stranded DNA resulted in markedly increased internalization compared to V66 antibody alone (Fig. S4B).

To test in vivo our hypothesis that V66-mediated delivery efficiency increases over time with treatments that enhance exDNA accumulation in the tumor microenvironment, we conducted an experiment in DLD1 tumor-bearing mice. One group was pre-treated with 10 Gy of radiation for two consecutive days to “prime” the tumors by inducing DNA damage. On day 3, both groups were administered a final dose of 100 µg of fluorescently labeled V66 (Fig. 4F). Tumors were excised 24 h later and imaged with IVIS system to assess the impact of radiation on V66 delivery and internalization. Fluorescence imaging revealed a significant marked increase in V66 accumulation in tumors “primed” with radiation compared to the group that did not receive the radiation (Fig. 4G), further supporting the hypothesis that DNA damage enhances the internalization pathway.

Together, these findings suggest that V66 antibody internalization relies on the accumulation of exDNA, which enhances its interaction with ENT2 transporters, rather than resulting from alterations in ENT2 expression itself.

### V66-exatecan crosses the BBB and effectively targets DDR-deficient Medulloblastoma

Medulloblastoma is the most prevalent malignant brain tumor in pediatric patients, and it is often linked to inherited mutations in genes that regulate DNA repair and replication stress mechanisms [51]. Recently, Lu et al. experimentally demonstrated that the GFAP-Cre mediated conditional loss of the genes *Brca1* or *Brca2* can induce spontaneous medulloblastoma formation in mice [34]. Using this innovative autochthonous model, we assessed the efficacy of V66-exatecan ADC in treating these challenging tumors, which require the ADC to cross the BBB.

**Fig. 5:**
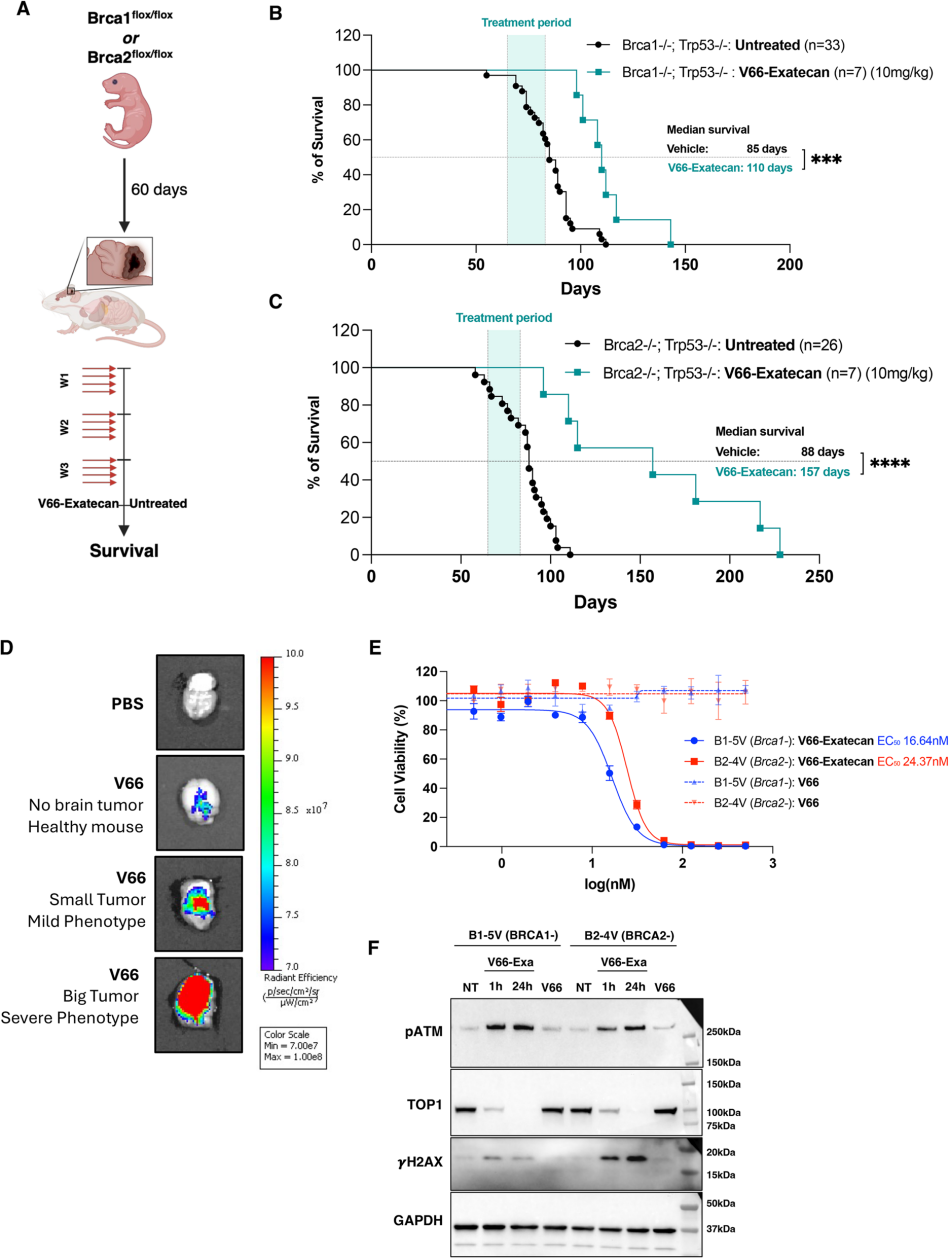
V66-exatecan successfully crosses the BBB and localizes to brain tumors, enhancing survival in two different DDR-deficient spontaneous medulloblastoma mouse models. **A** Schematic of efficacy studies using GEMMs forming tumors that are BRCA1 or BRCA2 deficient. Mice received 10 mg/kg of V66-exatecan by intraperitoneal injection on 4 consecutive days a week for 3 weeks. **B-C** Kaplan-Meier survival curves of conditional Brca1 (**B**) and Brca2 (**C**) KO mice. The genotypes and number of mice (n) in each of the GEMMs are shown by the labels. Median survival indicated on the plot. Statistical analysis was performed using the Log-rank (Mantel-Cox) test (****P < 0.0001). **D** Ex vivo IVIS imaging of brains from a genetically engineered mouse model (GEMM) of BRCA2-mutant medulloblastoma following administration of dye-labeled V66. Mice received 100 µg of VivoTag-680-labeled V66 via retro-orbital injection. Brains were harvested 24 h post-injection and imaged to assess V66 biodistribution. A PBS-injected control and a non-tumor-bearing mouse injected with labeled V66 were included to evaluate background signal. Increased V66 accumulation correlated with tumor burden, ranging from mild to severe phenotypes. **E** Dose-response curve showing the cytotoxicity of V66-exatecan in B1-5 V BRCA1-deficient cell lines (blue) and B2-4 V BRCA2-deficient cell lines (red). Cells were treated with V66-exatecan ADC for 24 h, and cell viability was assessed 7 days post-treatment using the CellTiter-Glo detection kit. Dashed lines in the same colors indicate cells treated with the V66 antibody alone, showing no observed toxicity in either cell line. Error bars represent the standard deviation of triplicate measurements (data are represented as mean ± SEM, *n* = 3). **F** Western blot analysis of B1-5 V (BRCA1-) and B2-4 V (BRCA2-) cells treated with PBS (NT, no treatment), V66-exatecan ADC for 1–24 h, or V66 alone (24 h). In samples treated with V66-exatecan ADC, a time-dependent accumulation of DNA damage is observed, as indicated by the increased γH2AX and pATM signals, along with a time-dependent down-regulation of TOP1

Mice with the Brca1^−/−^;Trp53^−/−^ or Brca2^−/−^;Trp53^−/−^ deletions were monitored until day 60 post-birth, at which point medullary tumors had developed. Starting on day 60, the mice were treated with V66-exatecan ADC, administered four times per week for three consecutive weeks, and subsequently monitored for survival (Fig. 5A). Treatment with V66-exatecan demonstrated significant efficacy in improving survival in the medulloblastoma mouse models. In Brca1^−/−^; Trp53^−/−^ mice, the median survival was extended from 85 days in the vehicle or untreated group to 110 days following V66-exatecan treatment (***P < 0.001, log-rank (Mantel-Cox) test) (Fig. 5B). The effect was even more pronounced in Brca2-/-; Trp53-/- mice, where treatment with the V66-exatecan ADC nearly doubled the median survival, extending it from 88 to 157 days (****P < 0.0001, log-rank [Mantel-Cox] test) (Fig. 5C).

Next, to further probe the ability of the V66 antibody to cross the blood-brain barrier (BBB) and selectively target brain tumors in the context of the autochthonous Brca2-/-; Trp53-/- tumors, tumor-bearing mice were injected with fluorescently labeled V66 antibody compared with tumor-bearing mice injected with PBS and with non-tumor-bearing mice injected with labeled V66 (to establish background signal in healthy brain tissue). Brains were excised 24 h post-injection and imaged for fluorescence. As shown in Fig. 5D, the tumor-bearing mice injected with fluorescently labeled V66 antibody displayed a clear, tumor-specific signal in the brain, whereas there was minimal signal in non-tumor bearing mice, providing direct evidence of V66 penetration into an autochthonous mouse brain tumor model that has a physiologic blood brain barrier. Further, signal intensity correlated with tumor burden, with stronger fluorescence observed in the mouse exhibiting more severe neurological symptoms corresponding to larger tumor size. No signal was seen with just PBS injection as expected.

We also investigated in vitro sensitivity to the V66-exatecan ADC in both Brca1^−/−^; Trp53^−/−^ and Brca2^−/−^; Trp53^−/−^ medulloblastoma-derived cells. Cells were treated with increasing concentrations of V66-exatecan ADC for 24 h, and their viability was assessed after 7 days. The results demonstrated remarkably low and comparable EC_50_ values, measured at ~ 16.64 nM for Brca1^−/−^ cells and ~ 24.37 nM for Brca2^−/−^ cells. In contrast, treatment with the unconjugated V66 antibody at any concentration had no impact on cell viability, confirming that the observed effects were specific to the payload of the ADC (Fig. 5E). Cells were also evaluated for effective activation of the DDR pathway. The Brca1^−/−^ and Brca2^−/−^ cells were treated with 100 nM of V66-exatecan ADC for 1–24 h, or with the unconjugated antibody for 24 h, and analyzed via western blotting. We found a time-dependent activation of the DDR pathway exclusively in samples treated with V66-exatecan ADC, as indicated by increased levels of γH2AX and phosphorylated ATM (Fig. 5F). Interestingly, γH2AX levels were higher in Brca2^−/−^ cells compared to Brca1^−/−^ cells, correlating with the extended survival observed in vivo for Brca2^−/−^ tumor-bearing mice. Additionally, the on-target activity of the ADC was validated by the time-dependent degradation of TOP1, confirming the efficacy of the conjugate.

These findings underscore the therapeutic potential of V66-exatecan in targeting medulloblastomas with DDR deficiencies, as found in patient cohorts, demonstrating its capacity to effectively cross the BBB, induce robust DDR activation, and significantly extend survival in preclinical models.

### V66-exatecan induces the DDR and inhibits tumor proliferation with a favorable toxicology profile

**Fig. 6:**
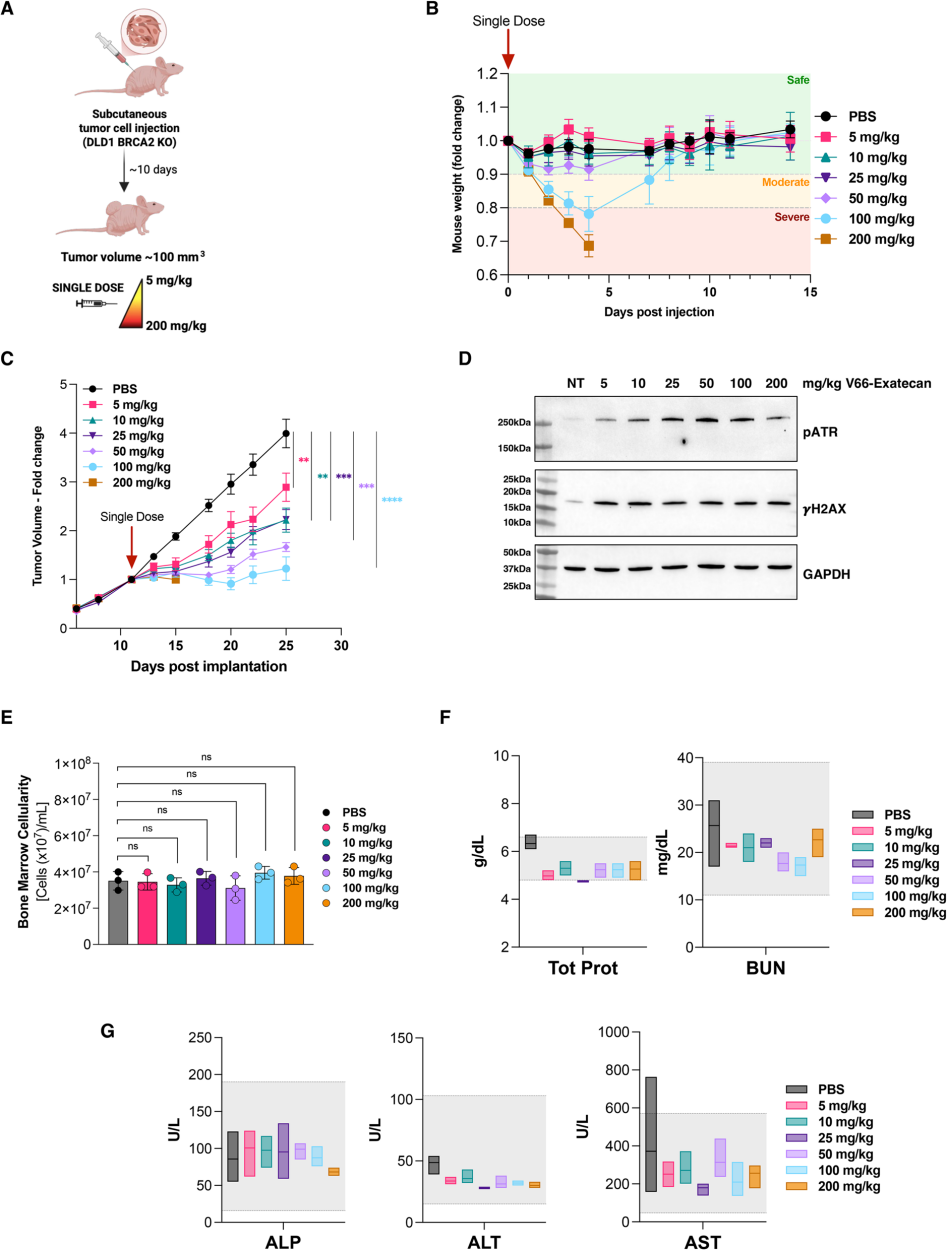
Favorable toxicology profile and therapeutic efficacy of V66-exatecan: single-dose administration activates DDR and inhibits tumor proliferation without hepatorenal toxicity. **A** Schematic of a comprehensive toxicology study involving a single intraperitoneal injection of V66-exatecan at increasing doses, ranging from 5 mg/kg to 200 mg/kg (*n* = 6 per group). **B** Body weight over time of mice from the study in panel (A). Treatments were well-tolerated at doses from 5 to 50 mg/kg (green area), mice that received 100 mg/kg showed signs of mild toxicity with subsequent recovery (yellow area). At the 200 mg/kg dose, mice exhibited significant weight loss (red area) and severe morbidity due to drug toxicity, leading to euthanasia of this group. **C** Tumor measurements of mice from the study in panel (A). One single dose of V66-exatecan suppresses cancer tumor growth in a dose-dependent manner. The plot shows tumor volumes in mice bearing DLD1 BRCA2KO subcutaneous xenograft flank tumors before and after injection of V66-exatecan (*n* = 6 per group). **D** Representative western blot of tumors collected from the study shown in panel (A). Tumors were collected 48 h post-injection, and total protein extracts were analyzed to assess activation of the DDR pathway, as indicated by the presence of γH2AX and pATM signals (*n* = 3). **E** Bone marrow cell counts of mice treated with increasing doses of V66-exatecan from the study shown in panel A (*n* = 3). **F-G** Evaluation of hematological and biochemical markers in mouse serum 48 h after treatment with PBS or various single doses of V66-exatecan. Following sacrifice, plasma activity levels were assessed for: (**F**) total protein (Tot Prot) and kidney-specific blood urea nitrogen (BUN), and (**G**) liver-specific enzymes alanine transaminase (ALT) and aspartate transaminase (AST) along with the bone-related enzyme alkaline phosphatase (ALP). Gray area on the plot indicate the normal concentration range for each marker

Exatecan showed promising efficacy in several clinical trials, but its potential was compromised by significant myelosuppression and severe gastrointestinal toxicity, which ultimately restricted its further development [52, 53]. To evaluate the in vivo toxicity in comparison to anti-tumor efficacy of V66-exatecan ADC, we tested the effects of single doses ranging from 5 mg/kg to 200 mg/kg. We used DLD1 BRCA2KO cells to form tumors subcutaneously in the flanks of nude mice, and following tumor establishment, we administered a single injection of V66-exatecan ADC at different doses: 5, 10, 25, 50, 100, and 200 mg/kg (*n* = 6) (Fig. 6A). This approach aimed to determine the threshold for toxicity from a single dose (maximum tolerated dose) and to simultaneously evaluate the efficacy of single doses as low as 5 mg/kg and as high as 200 mg/kg. Following treatment, mice were monitored for survival and changes in weight and behavior, and a subset of mice was sacrificed for detailed blood and bone marrow analyses. The results of the single-injection dose-escalation study for V66-exatecan ADC demonstrated a dose-dependent effect on mouse weight and health (Fig. 6B). Mice injected with lower doses (5–25 mg/kg) showed no weight loss, while those injected with 50 mg/kg experienced minimal, safe and transient weight loss, recovering to baseline levels within a few days. At the higher dose of 100 mg/kg, moderate weight loss was observed, followed by gradual recovery over time. Mice in the highest dose group (200 mg/kg) exhibited severe adverse effects, including significant weight loss and gastrointestinal distress. These toxicities necessitated the early termination of the study for the 200 mg/kg group.

The efficacy of a single-dose injection of V66-exatecan ADC was also assessed by monitoring tumor volume over time across the different dose groups following the single treatment (Fig. 6C). Even at the lowest dose tested (5 mg/kg group), tumor growth was reduced, showing a significantly slower growth compared to the control group. As the dose increased, there was more effective tumor growth inhibition, with a more noticeable slowdown in tumor progression in the 10 and 25 mg/kg groups compared to the 5 mg/kg group or the control. However, the tumor volumes in these groups resumed growth at 5 to 10 days after treatment. At the higher single doses (50–100 mg/kg), significant and more durable tumor growth inhibition was observed, with some tumors shrinking or stabilizing in size (*n* = 6 per group) (**P < 0.01, ***P < 0.001, ****P < 0.0001 two-way ANOVA multiple comparison test). In the 200 mg/kg group, while initial tumor growth inhibition was observed, the study had to be terminated after day 4 due to severe toxicity. To evaluate the induction of DNA damage following the single-dose injection, we interrogated the activation of the DDR pathway in vivo. Tumors were collected 48 h post-injection of the single doses, and proteins were extracted for analysis. Western blotting revealed substantial induction of DNA damage, as evidenced by the increased expression of histone γH2AX and phosphorylation of ATR (pATR) (*n* = 3) (Fig. 6D).

Additionally, as a measure of toxicity, bone marrow cellularity was analyzed 48 h post-treatment by collecting bone marrow from the femurs and tibias of mice. No significant changes in bone marrow cellularity were observed between the untreated control and single-dose treatment groups (*n* = 3) (Fig. 6E). Finally, we collected blood samples from the mice to assess kidney and liver function by measuring key biomarkers. Kidney function was evaluated by analyzing total protein levels and blood urea nitrogen (BUN) (Fig. 6F), while liver function was assessed by measuring alkaline phosphatase (ALP), alanine aminotransferase (ALT), and aspartate aminotransferase (AST) (Fig. 6G). The results showed no significant changes in any of these parameters between the treated and control groups, suggesting that the single-dose treatment did not induce noticeable alterations in kidney or liver function in the short term.

To examine the toxicity profile of V66-exatecan ADC more thoroughly, we conducted both short- and long-term toxicity evaluations in a separate group of mice bearing DLD1 BRCA2KO tumors, which underwent the full 3-week treatment regimen previously used for the efficacy study (as in Fig. 3D). These mice were administered either two or four doses per week over a three-week period of V66-exatecan ADC or exatecan mesylate (equimolar dose, 0.26 mg/kg). After completing the treatment, we performed a short-term toxicity evaluation 7 days post-treatment, measuring the complete blood count in mice from the vehicle control, 2X, and 4X treatment groups. The experiment showed no significant differences in white blood cell counts (WBC), hemoglobin levels (Hb), or red blood cell (RBC) counts between mice treated with V66-exatecan ADC or exatecan mesylate compared to the vehicle group, regardless of whether they received 2X or 4X doses (Fig. S5A). Moreover, mice that received three weeks of V66-exatecan ADC treatment were further evaluated for potential long-term toxicity to muscle, kidney, or liver 30 days post-treatment. Histological sections of the quadriceps and heart, stained with H&E, showed no signs of toxicity in mice treated with V66-exatecan ADC twice or four times per week (Fig. S5B). Additionally, blood samples were analyzed for creatine phosphokinase and myogenin levels, with no significant changes observed compared to the vehicle group (Fig. S5C). Liver function was assessed by measuring ALP, ALT, and AST, while kidney function was evaluated by analyzing total protein and BUN levels, with no significant differences observed compared to the vehicle group (Fig. S5D-E).

Overall, the above results demonstrate dose-dependent efficacy of V66-exatecan ADC, with significant tumor growth inhibition observed at higher doses. Short- and long-term toxicity assessments showed no significant changes in hematology, muscle, liver, or kidney function. These findings indicate that V66-exatecan ADC is effective with minimal toxicity, supporting its potential for further therapeutic development.

## Discussion

While ADCs have shown considerable progress, their broader clinical application is still hindered by key challenges. The limited pool of tumor-specific antigens available for targeted therapy, coupled with the low internalization efficiency of the ADCs, reduces their therapeutic potential and restricts their effectiveness across diverse cancer types [9, 14]. This study highlights the promising potential of the humanized V66 anti-DNA antibody, addressing the limitations of conventional ADCs. Our findings uncover a complex dual-targeting mechanism, where the accumulation of exDNA and overexpression of ENT2 transporters direct the V66 antibody to the tumor site, enabling it to penetrate the cell nuclei. This mechanism sets V66 apart from other ADCs, requiring the presence of two components that are widely present across diverse tumor types but are minimally found in healthy tissues, rather than relying on single surface antigens. As a result, V66 has the potential to overcome some of the limitations of traditional ADCs and may offer a novel therapeutic strategy.

Here, we demonstrate that the humanized V66 anti-DNA antibody selectively targets tumors and achieves nuclear localization through a mechanism that depends on both exDNA and ENT2 and is independent of canonical internalization pathways such as endosomal-lysosomal trafficking. In vivo studies confirmed specific tumor accumulation and minimal off-target uptake, with the exception of the liver. In previous studies [26], we observed that the liver uptake of the 3E10 class of antibodies occurs primarily in non-parenchymal cells and not hepatocytes consistent with known antibody clearance mechanisms in the liver. Consistent with this, we do not see liver toxicity in the treated mice. This high tumor selectivity of V66 makes it an ideal candidate for an ADC approach. We conjugated the V66 antibody with exatecan, a synthetic derivative of camptothecin and potent TOP1 inhibitor, whose promising efficacy as a monotherapy is constrained by dose-limiting toxicities [52, 53]. In vivo experiments confirmed that the conjugation did not alter the biological properties of V66, with the ADC primarily localizing to the nucleus in tumor cells in vitro and in vivo. Moreover, in vitro testing across a range of cancer cell lines demonstrated robust anti-tumor activity at low nanomolar concentrations, underscoring the promising therapeutic potential of the V66-exatecan ADC for targeting diverse tumor types. Notably, non-tumor primary mouse fibroblasts exhibited no signs of toxicity under the same treatment conditions, highlighting the selectivity of the ADC for malignant cells.

V66-exatecan ADC exerts its cytotoxic effects through targeted nuclear delivery and subsequent release of exatecan, which inhibits TOP1, induces DNA damage, and activates DDR pathways that ultimately lead to cell death. We explored the mechanisms underlying exatecan release and found that it can occur either through cleavage by Cathepsin B or via proteasomal degradation. In addition, the V66-exatecan ADC demonstrated a notable bystander effect, likely due to the membrane-permeable nature of exatecan, which enables the drug to diffuse into neighboring, untreated cells and contribute to enhanced anti-tumor activity.

In particular, V66-exatecan demonstrated exceptional efficacy against challenging tumors, such as TNBC, a highly aggressive subtype of breast cancer. TNBC is notoriously difficult to treat due to its lack of hormone receptors and HER2 expression, which makes it unresponsive to targeted therapies, along with its immunosuppressive tumor microenvironment and high metastatic potential. The efficacy of V66-exatecan in TNBC was confirmed in cell culture through the induction of DNA damage and the down-regulation of the target enzyme, TOP1, central to the therapeutic action of the ADC. In vivo, using TNBC xenografts, V66-exatecan demonstrated strong, dose-dependent suppression of tumor growth, with evident tumor regression and significant survival benefits compared to control treatments, showcasing a substantial survival advantage. Remarkably, the treatment was well-tolerated, with no signs of toxicity observed, suggesting its potential for further clinical development.

The current study also highlights the enhanced efficacy of V66-exatecan in tumors with BRCA1/2 deficiencies, where these mutations significantly amplify the therapeutic potential of the ADC payload. *BRCA1/2* are crucial tumor suppressor genes that mediate DNA repair through homologous recombination, and their loss leads to a diminished capacity for DNA repair, rendering these tumors highly sensitive to DNA-damaging agents [47]. Our results show that BRCA1/2-deficient tumors are markedly more sensitive to V66-exatecan, with in vitro assays revealing up to a 17-fold increase in sensitivity compared to BRCA2-proficient cells. This heightened sensitivity also correlates with a more pronounced internalization of the V66 antibody in DDR-deficient cells, due to the availability of more exDNA in the tumor microenvironment. This observation supports findings by Rackear et al., who demonstrated that the targeting capacity of V66 is directly related to its high affinity for nucleic acids [19], further emphasizing the critical role of exDNA in enhancing antibody specificity and uptake.

We demonstrated, both in vitro and in vivo, that the accumulation of exDNA in the tumor microenvironment plays a pivotal role in enhancing antibody uptake and tumor targeting. Notably, in vivo experiments revealed that “priming” tumors with radiation before V66-exatecan treatment significantly increased antibody internalization, thereby amplifying its therapeutic effect. This finding highlights the potential of combination strategies to optimize treatment efficacy, further emphasizing the critical role of exDNA in modulating drug delivery and reinforcing the therapeutic advantage of V66-exatecan in BRCA1/2-deficient tumors.

In vivo, V66-exatecan ADC exhibited remarkable efficacy against BRCA2-deficient xenografts, with significant tumor regression observed in treated animals. A notable proportion of the animals even achieved complete regression at the end of the study, highlighting the substantial survival benefit afforded by this treatment. This potent therapeutic effect underscores the potential of V66-exatecan to exploit the vulnerabilities inherent in BRCA1/2-deficient tumors, offering a promising strategy for treating cancers driven by these mutations.

Moreover, these findings are further validated in the context of central nervous system (CNS) malignancies, where the BBB has traditionally posed a major challenge for drug delivery, including most ADCs. Notably, V66-exatecan demonstrated the ability to cross the BBB, effectively targeting BRCA1- and BRCA2-deficient autochthonous medulloblastomas. In vivo studies showed efficient tumor targeting and significant survival benefits, with the most pronounced therapeutic effect observed in BRCA2-deficient tumors. These results not only emphasize the capability of V66-exatecan to address tumor-specific vulnerabilities in a range of cancer types but also highlight its potential as a transformative approach for treating previously hard-to-reach CNS tumors. Notably, these promising results were achieved while maintaining a significant safety margin, as V66-exatecan ADC remained well below toxicity thresholds even at relatively high doses, highlighting its potential for further development and possible clinical application.

As V66-exatecan progresses through further development and optimization, it offers potential for overcoming the treatment challenges posed by difficult-to-target and historically inaccessible tumors and provides the basis for a new class of ADCs.

## Conclusion

V66-exatecan represents a next-generation ADC that addresses critical limitations of traditional platforms by leveraging exDNA-mediated tumor selectivity and ENT2-facilitated nuclear delivery of its cytotoxic payload. This dual-targeting strategy enables precise tumor engagement and efficient drug release, resulting in potent anti-tumor activity across multiing survival while maintaining high tolerabiliple models. Its broad therapeutic efficacy, combined with an excellent safety profile, underscores its promise as a targeted treatment for DDR-deficient cancers and anatomically challenging or poorly accessible tumors.

## Supplementary Information

Supplementary Material 1.

## Data Availability

Data is provided within the manuscript or supplementary information files.

## Abbreviations

ADC: Antibody-drug conjugate
ALP: Alkaline phosphatase
ALT: Alanine aminotransferase
AST: Aspartate aminotransferase
BBB: Blood-brain barrier
BRCA1/2: Breast cancer gene 1 and 2
BUN: Blood urea nitrogen
CatB: Cathepsin B
CDR: Complementarity-determining region
CNS: Central nervous system
DAR: Drug-to-antibody ratio
DDR: DNA damage response
EC50: Half maximal effective concentration
ENT2: Equilibrative nucleoside transporter 2
exDNA: Extracellular DNA
GEMM: Genetically engineered mouse model
Hb: Hemoglobin
HPLC: High-performance liquid chromatography
PBS: Phosphate-buffered saline
PCR: Polymerase chain reaction
RBC: Red blood cell
RT: Room temperature
TNBC: Triple-negative breast cancer
TOP1: Topoisomerase I
WBC: White blood cell
WT: Wild type
