# Supplementary material for: Harnessing exDNA for precision exatecan delivery in cancer: a novel antibody-drug conjugate approach
Source: Mol Cancer. 2025 Oct 13;24:253. doi: 10.1186/s12943-025-02462-z (PMC12516839; doi:10.1186/s12943-025-02462-z)
Supplement: Supplementary file 1 — Supplementary Material 1. [file 12943_2025_2462_MOESM1_ESM.pdf]

Supplementary Figure 1

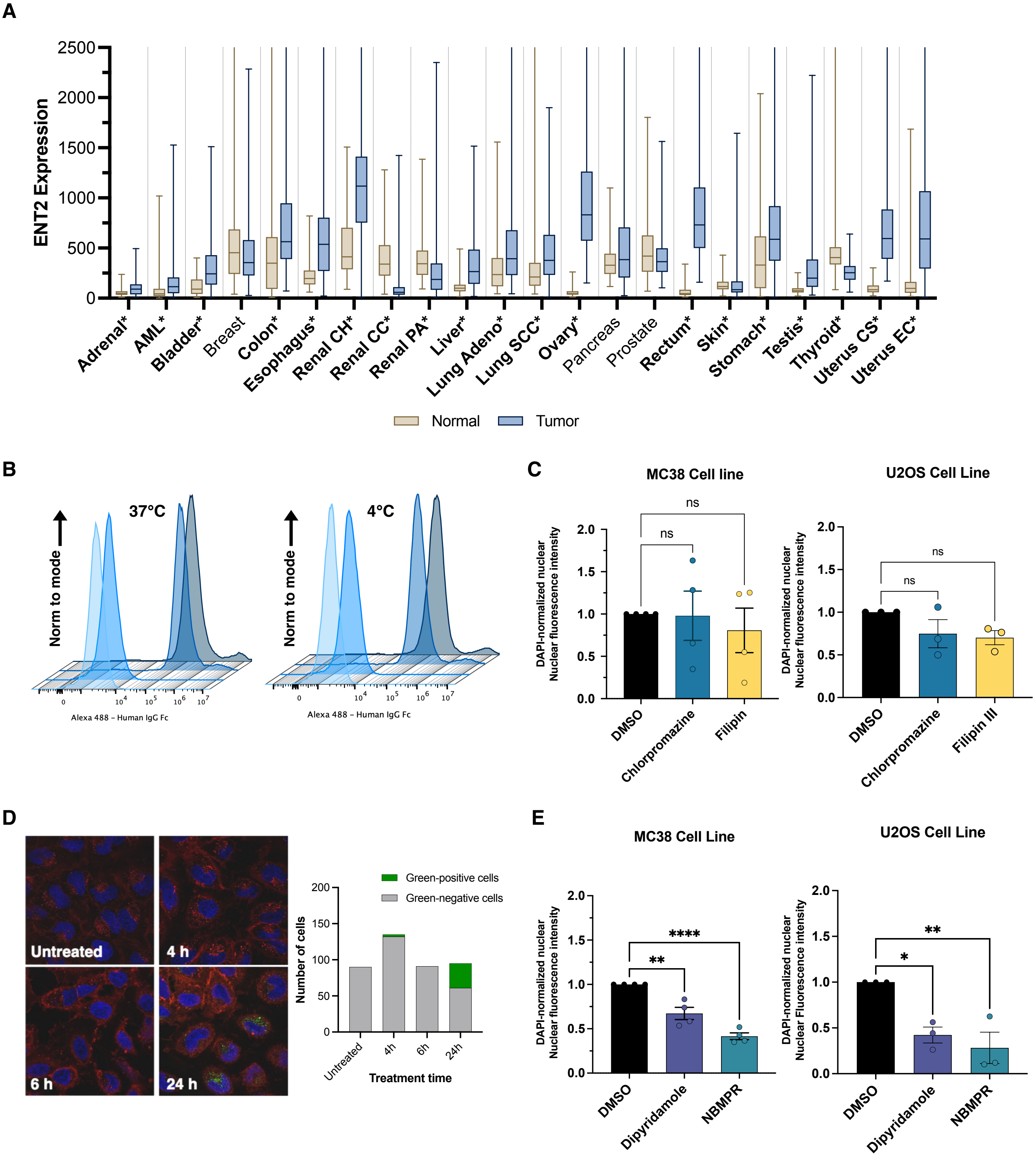

Suppl. Fig. 1. Cellular uptake mechanisms of V66 antibody. (**A**) Comparison of ENT2 mRNA expression levels (measured as Fragments Per Kilobase of exon per Million reads) in normal and oncogenic human tissue samples. Data were obtained from The Cancer Genome Atlas Project (NCI) and accessed via TNMplot.com. (**B**) Endocytosis involvement in 3E10 antibody uptake was tested in K562 cells pre-incubated at 37 °C or 4 °C for 45 minutes, followed by 1 µM treatment with chimeric 3E10-D31N or humanized high-affinity V66 for 1 hour. Cells were fixed, permeabilized, stained with anti-human Fc secondary antibody, and analyzed by flow cytometry. PBS and IgG1 isotype were used as controls. Uptake was unaffected by low temperature, suggesting endocytosis is not required. (**C**) V66 uptake is not affected by chemical inhibition of endocytosis. MC38 (left) and U2OS (right) cells were pre-treated with chlorpromazine (clathrin-mediated endocytosis inhibitor) or filipin III (caveolae-mediated endocytosis inhibitor) for 30 min, followed by co-treatment with 1 µM V66 for 1 h. Cells were fixed, permeabilized, stained with anti-human Fc secondary antibody, and analyzed by immunofluorescence imaging. Data represent 3–4 biological replicates with $n \geq 100$ cells per replicate. Statistical significance was determined by one-way ANOVA (n.s., not significant). (**D**) V66 was conjugated to Zenon pHrodo IgG dye, which fluoresces green in acidic environments such as lysosomes. HeLa cells were treated with 1 µM pHrodo-conjugated V66 for the indicated times, then fixed, stained, and analyzed by immunofluorescence imaging. Cells were classified as green-positive or green-negative based on lysosomal accumulation, and quantification is shown on the right. (**E**) MC38 cells (left) and U2OS cells (right) were pre-treated with the ENT2 inhibitors Dipyridamole or S-(4-Nitrobenzyl)-6-thioinosine (NBMPR) for 30 minutes. Following this, cells were incubated with the inhibitors plus 1 µM V66 for 1 hour, then fixed, permeabilized, and stained with a secondary antibody against the human Fc. Immunofluorescence imaging was performed to assess V66 cellular penetration. Data represent 3–4 biological replicates with at least 100 cells analyzed per replicate. Statistical significance was determined using one-way ANOVA. (* $P<0.05$, ** $P<0.01$, **** $P<0.0001$).

Supplementary Figure 2

A

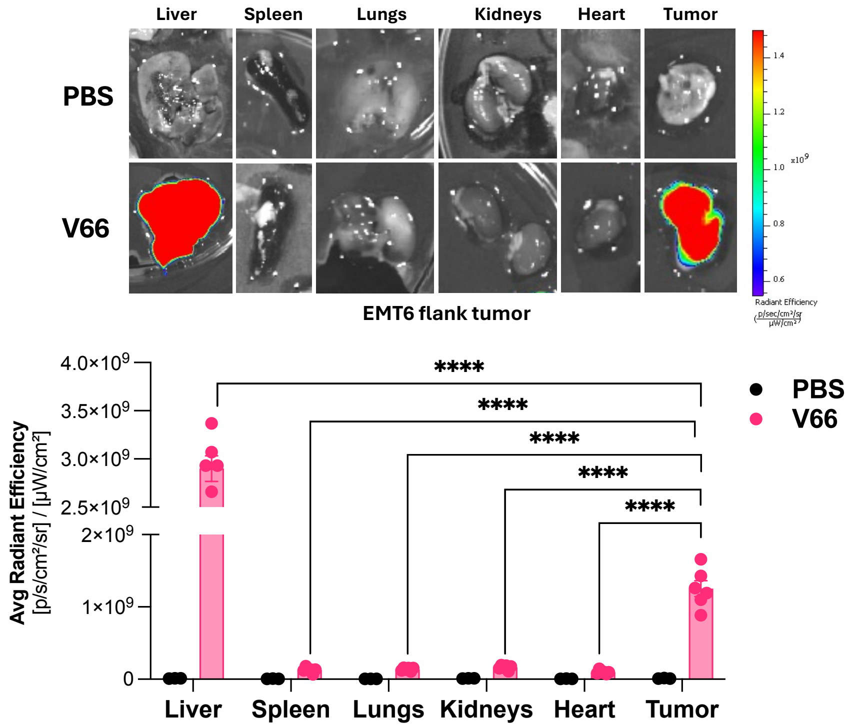

B

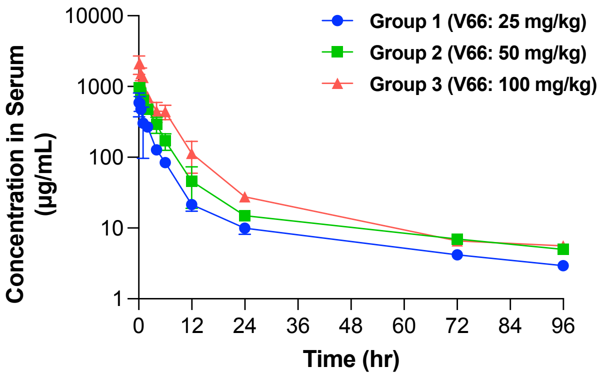

Suppl Fig. 2. Biodistribution and Pharmacokinetics of V66. (**A**)Representative IVIS images of tissues collected from a biodistribution experiment in Balb/c mice with subcutaneous EMT6 syngeneic tumors. Mice received 100 µg of dye-labeled V66 or PBS, and tissues were harvested 24 hours post-treatment. Relative quantification is shown in the lower panel. (****P < 0.0001, Student's t-test) (n=3-5). (**B**) Serum concentration-time profile of V66 following a single intravenous (IV) administration at doses of 25 mg/kg, 50 mg/kg, and 100 mg/kg in mice (C57BL/6). Blood samples were collected at multiple time points (0, 5 min, 15 min, 30 min, 1-, 2-, 4-, 6-, 12-, 24-, 72-, and 96-hours post-dose), and serum antibody concentrations were quantified using ELISA-ECL based assay on MSD platform. Data are presented as mean ± standard deviation (SD) (n=3).

Supplementary Figure 3

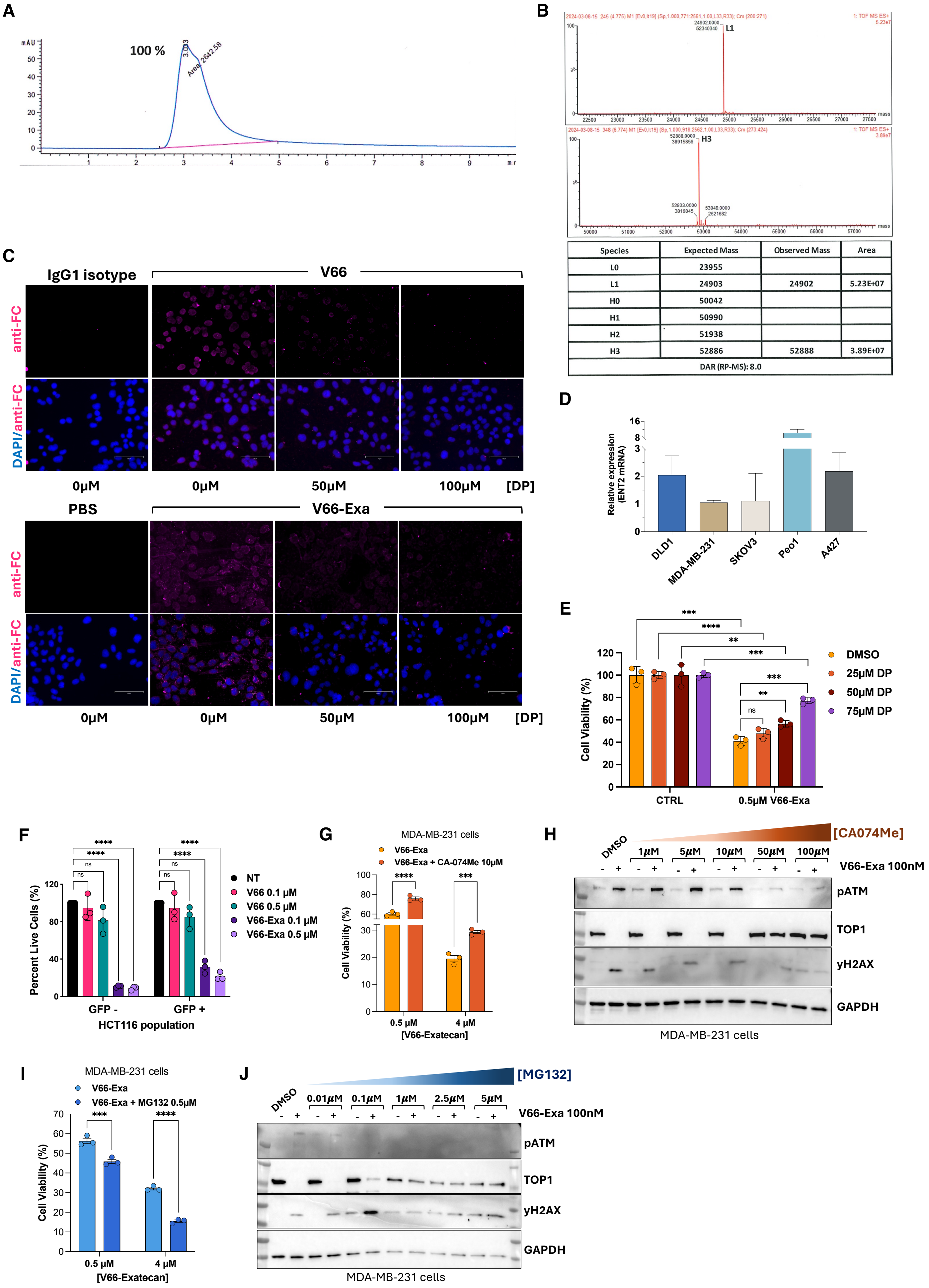

Species	Expected Mass	Observed Mass	Area
L0	23955		
L1	24903	24902	5.23E+07
H0	50042		
H1	50990		
H2	51938		
H3	52886	52888	3.89E+07
DAR (RP-MS): 8.0			

Suppl. Fig. 3. Purification and Drug-to-Antibody Ratio (DAR) determination of V66-Exatecan ADC. (**A**) To assess monomer purity, the ADC conjugate was analyzed by Size-Exclusion Chromatography (SEC) using an Agilent AdvanceBio SEC column (200 Å, 2.7 µm, 4.6 × 150 mm) with a mobile phase of 50 mM Sodium Phosphate and 100 mM NaCl (pH 6.3). SEC confirmed that the purified ADC exhibited 100% monomeric purity. (**B**) The Drug-to-Antibody Ratio (DAR) was determined by Reverse Phase Mass Spectrometry (RP-MS) on a PLRP-S column (1000 Å, 8 µm, 2.1 × 50 mm) using a Xevo G2-XS mass spectrometer, yielding a calculated DAR of 8. (**C**) Immunofluorescence imaging of DLD1 cells treated with PBS, V66, or IgG1 isotype control, alone or in combination with dipyridamole (50 or 100 µM). Images were acquired using an EVOS fluorescence microscope. (**D**) qRT-PCR analysis of ENT2 transporter expression in DLD1, MDA-MB-231, SKOV3, Peo1, and A427 cells (n = 3–4). ENT2 mRNA levels were normalized to ActB; no significant differences were observed among the cell lines. (**E**) DLD1 cells were pretreated for 1 h with DMSO or dipyridamole (25, 50, or 75 µM) before treatment with 0.5 µM V66-Exatecan to test ENT2 involvement. Cell viability was measured after 72 h using the CellTiter-Glo assay, showing that dipyridamole pretreatment did not significantly affect ADC. (**F**) Bystander killing was evaluated by treating HCT116 (GFP-) cells with 0.1 or 0.5 µM of antibody or ADC for 24 h, followed by co-culture with untreated HCT116 (GFP+) cells. GFP signal (flow cytometry, 48 h) indicated a strong bystander effect (n = 3). (**G**) MDA-MB-231 cells treated with 0.25 or 4 µM V66-Exatecan ± 10 µM Cathepsin B inhibitor CA074Me for 72 h showed partial rescue of viability, suggesting Cathepsin B–dependent cleavage. (**H**) Western blot of MDA-MB-231 cells treated with 100 nM V66-Exatecan ± CA074Me (1-100 µM) showed reduced DDR activation (γH2AX, pATM) and partial restoration of TOP1 at high, toxic inhibitor doses. (**I**) MDA-MB-231 cells were treated with 0.5 or 4 µM V66-Exatecan ± 0.5 µM MG132 for 72 h to evaluate proteasomal degradation. Cell viability (CellTiter-Glo) showed significant rescue upon MG132 treatment. (**J**) Western blot analysis of MDA-MB-231 cells treated with 100 nM V66-Exatecan ± MG132 (0.01-5 µM) revealed a dose-dependent reduction in DDR activation and partial rescue of TOP1 degradation at high, cytotoxic inhibitor concentrations. (**P < 0.01, ***P < 0.001, ****P $<$ 0.0001, Student's t-test).

Supplementary Figure 4

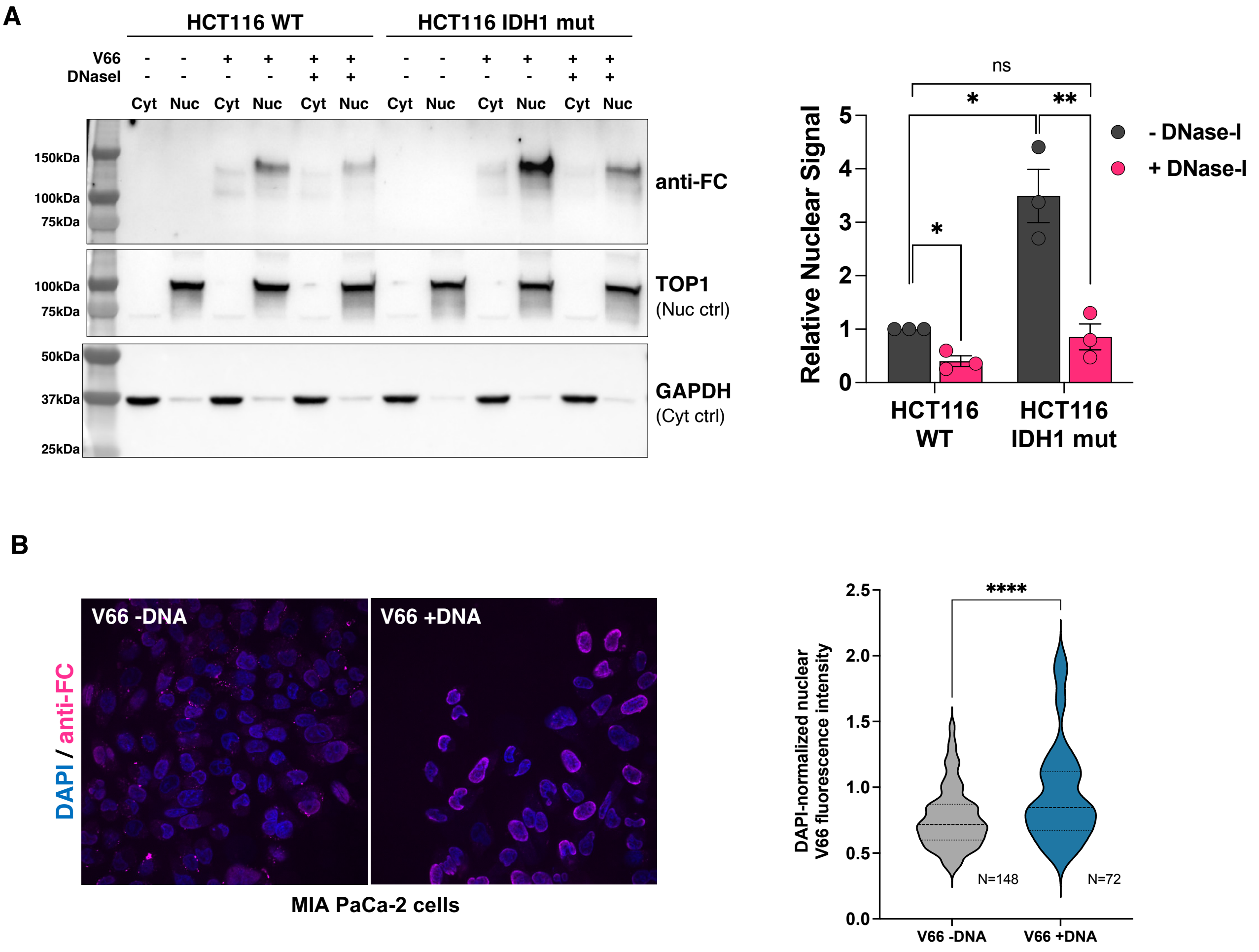

Suppl. Fig 4. Increased V66 penetration in DDR-mutant cells and its dependence on extracellular DNA (**A**) Representative western blot analysis demonstrating the dependence of V66 antibody penetration on extracellular DNA. HCT116 IDH1 mutant cells show greater internalization of V66 compared to DDR-proficient (WT) cells under normal conditions; pre-treatment with DNase-I significantly reduces V66 penetration in both WT and IDH1 mutant cells. Cells were pre-treated with DNase-I for 10 minutes, followed by 1 hour of V66 treatment. Relative quantification is shown in the right panel. Data are presented as mean ± SEM (n = 3) (* P<0.05, ** P<0.01, ns, not significant, Student's t-test). (**B**) VivoTag-680-labeled V66 antibody was incubated with double-stranded DNA at room temperature for 10 minutes to form non-covalent V66–nucleic acid complexes (25 µg V66 and 10 µg of dsDNA per reaction). MIA PaCa-2 cells were treated with 1 µM of the complexes for 1 hour, then fixed, permeabilized, and imaged by confocal immunofluorescence microscopy. A minimum of 150 cells were analyzed per group.). (**** P<0.0001, Student's t-test).

Supplementary Figure 5

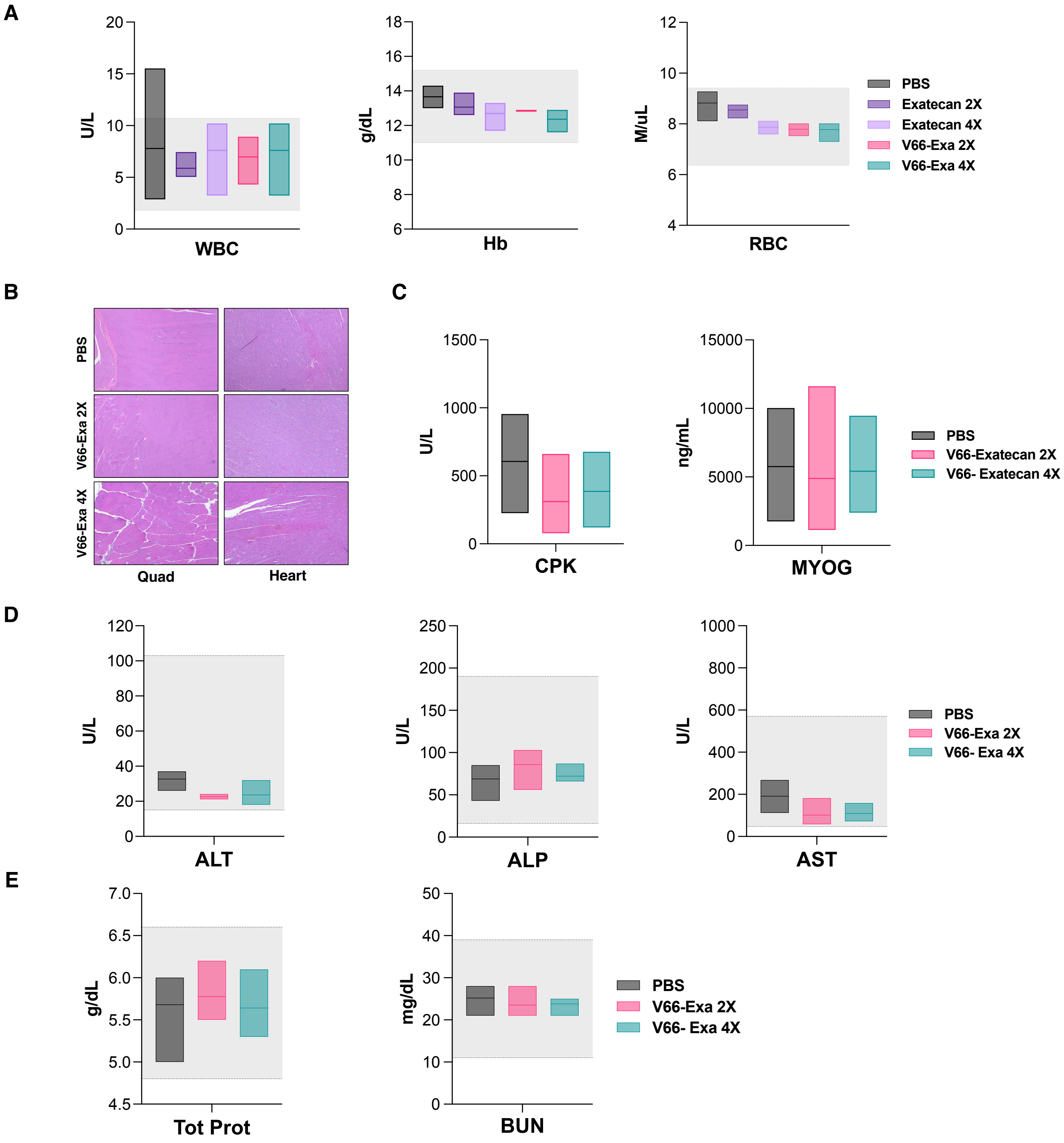

Suppl. Fig. 5. Evaluation of short- and long-term toxicity following V66-Exatecan ADC treatment. (**A**) Short-term toxicity evaluation in complete blood count (CBC) 7 days after the last injection of PBS, Exatecan Mesylate (2X and 4X) or V66-Exa ADC (2X or 4X). Blood samples were collected, and CBC analysis was performed to assess potential hematological changes. WBC, white blood cells; Hb, hemoglobin; RBC, red blood cells. (**B**) Representative images of hematoxylin and eosin (H&E) staining of quadriceps (left) and heart (right) 30 days post-treatment. No muscle damage was observed. n = 3. (**C-E**) Long-term evaluation of hematological and biochemical markers in mouse serum 30 days after the last injection of PBS or V66-Exa ADC (2X or 4X). Upon sacrifice, plasma activity levels were assessed for: (**C**) muscle-specific enzyme (MYOG) and muscle damage-related enzyme (CPK); (**D**) liver-specific enzymes (AST and ALT) and bone-related enzyme (ALP); (**E**) kidney-specific enzymes (BUN) and total protein (Tot Prot) (n = 5). ALP, alkaline phosphatase; ALT, alanine transaminase; AST, aspartate transaminase; BUN, blood urea nitrogen; Tot Prot, total protein; MYOG, myoglobin; CPK, creatine phosphokinase.
